# Revealing the True Incidence of Pandemic A(H1N1)pdm09 Influenza in Finland during the First Two Seasons — An Analysis Based on a Dynamic Transmission Model

**DOI:** 10.1371/journal.pcbi.1004803

**Published:** 2016-03-24

**Authors:** Mikhail Shubin, Artem Lebedev, Outi Lyytikäinen, Kari Auranen

**Affiliations:** 1 University of Helsinki, Helsinki, Finland; 2 National Institute for Health and Welfare, Helsinki, Finland; 3 Rybinsk State Aviation Technical University, Rybinsk, Russia; 4 University of Turku, Turku, Finland; The Pennsylvania State University, UNITED STATES

## Abstract

The threat of the new pandemic influenza A(H1N1)pdm09 imposed a heavy burden on the public health system in Finland in 2009-2010. An extensive vaccination campaign was set up in the middle of the first pandemic season. However, the true number of infected individuals remains uncertain as the surveillance missed a large portion of mild infections. We constructed a transmission model to simulate the spread of influenza in the Finnish population. We used the model to analyse the two first years (2009-2011) of A(H1N1)pdm09 in Finland. Using data from the national surveillance of influenza and data on close person-to-person (social) contacts in the population, we estimated that 6% (90% credible interval 5.1 – 6.7%) of the population was infected with A(H1N1)pdm09 in the first pandemic season (2009/2010) and an additional 3% (2.5 – 3.5%) in the second season (2010/2011). Vaccination had a substantial impact in mitigating the second season. The dynamic approach allowed us to discover how the proportion of detected cases changed over the course of the epidemic. The role of time-varying reproduction number, capturing the effects of weather and changes in behaviour, was important in shaping the epidemic.

## Author Summary

In 2009, the threat of the new pandemic influenza A(H1N1)pdm09 (referenced in media as ‘swine flu’) created a heavy burden to the public health systems wordwide. In Finland, an extensive vaccination campaign was set up in the middle of the first pandemic season 2009/2010. However, the true number of infected individuals remains uncertain as the surveillance missed a large portion of mild infections. We built a probabilistic model of influenza transmission that accounts for observation bias and the possible impact of the changing weather and population behaviour. We used the model to simulate the spread of influenza in Finland during the two first years (2009-2011) of A(H1N1)pdm09 in Finland. Using data from the national surveillance of influenza and data on social contacts in the population, we estimated that 9% of the population was infected with A(H1N1)pdm09 during the studied period. Vaccination had a substantial impact in mitigating the second season.

## Introduction

The threat of the pandemic influenza A strain, A(H1N1)pdm09 (‘swine flu’), imposed a huge burden on the public health system in Finland in 2009 [1]. The first A(H1N1)pdm09 season was part of the global pandemic and occurred from September 2009 through January 2010 with a major outbreak in November 2009. To mitigate the epidemic, a national vaccination campaign was started in October 2009, and by February 2010 approximately half of the Finnish population had been vaccinated against A(H1N1)pdm09. The second epidemic season occurred a year later from November 2010 through April 2011. Only sporadic cases were observed before the first epidemic season and between the two seasons.

It is well known that that laboratory-based surveillance of influenza misses the vast majority of infections that occur in the population. Underreporting follows from asymptomatic or non-diagnosed infection or incomplete reporting of influenza cases in primary and secondary health care. More severe cases are diagnosed and reported with a higher probability. This was true also for A(H1N1)pdm09, although special efforts were taken to record cases especially during the early phases of the first season.

Bayesian methodology (evidence synthesis) has been used to analyse influenza outbreaks in the presence of underreporting and mixed data sources. In general, the underlying epidemiological models can be classified as static or dynamic. In static models, cases are typically aggregated by season and the unknown true incidence is estimated as an attack rate (probability of becoming infected during the season) [2–5]. In dynamic models, the process of spread of the infection via transmission is modelled explicitly [6]. The static approach is simpler and requires less computational resources while the dynamic model enables one to answer more complex questions.

Based on a static model, we previously estimated that only 4% of the Finnish population were infected with A(H1N1)pdm09 over the season 2009/2010 and an additional 1% during the 2010/2011 season [2]. The most affected age groups were children and teenagers with attack rates up to 10-12%. The attack rates were much lower in the second season, which was likely due to the relatively high immunity due to natural infection or vaccination in the most influential age groups. In particular, 74-81% of children aged less than 15 years had been vaccinated against A(H1N1)pdm09 before the second season.

However, a static model cannot address the impact of herd immunity induced by vaccination. To properly address the role of vaccination in mitigating the first-season epidemic and lowering the transmission potential before the second season, a more dynamic (i.e. transmission) model is needed. A dynamic model can also address questions about which age groups played the most important role in transmission or why there was a second season despite the fact the influenza strain did not evolve considerably between the seasons to escape population immunity [7]. The effect of time-varying conditions due to weather or public response to the outbreak can also be inferred using a dynamic model [8].

In this study, we built a dynamic probabilistic model of influenza transmission and disease. The model accounts for transmission of influenza in the population, the impact of vaccination, outcomes with varying severity and imperfect detection of infection. We calibrate the model to data on A(H1N1)pdm09 cases and estimate the true incidence of A(H1N1)pdm09 of the first two A(H1N1)pdm09 seasons in Finland.

## Methods

### Data sources

**Fig 1:**
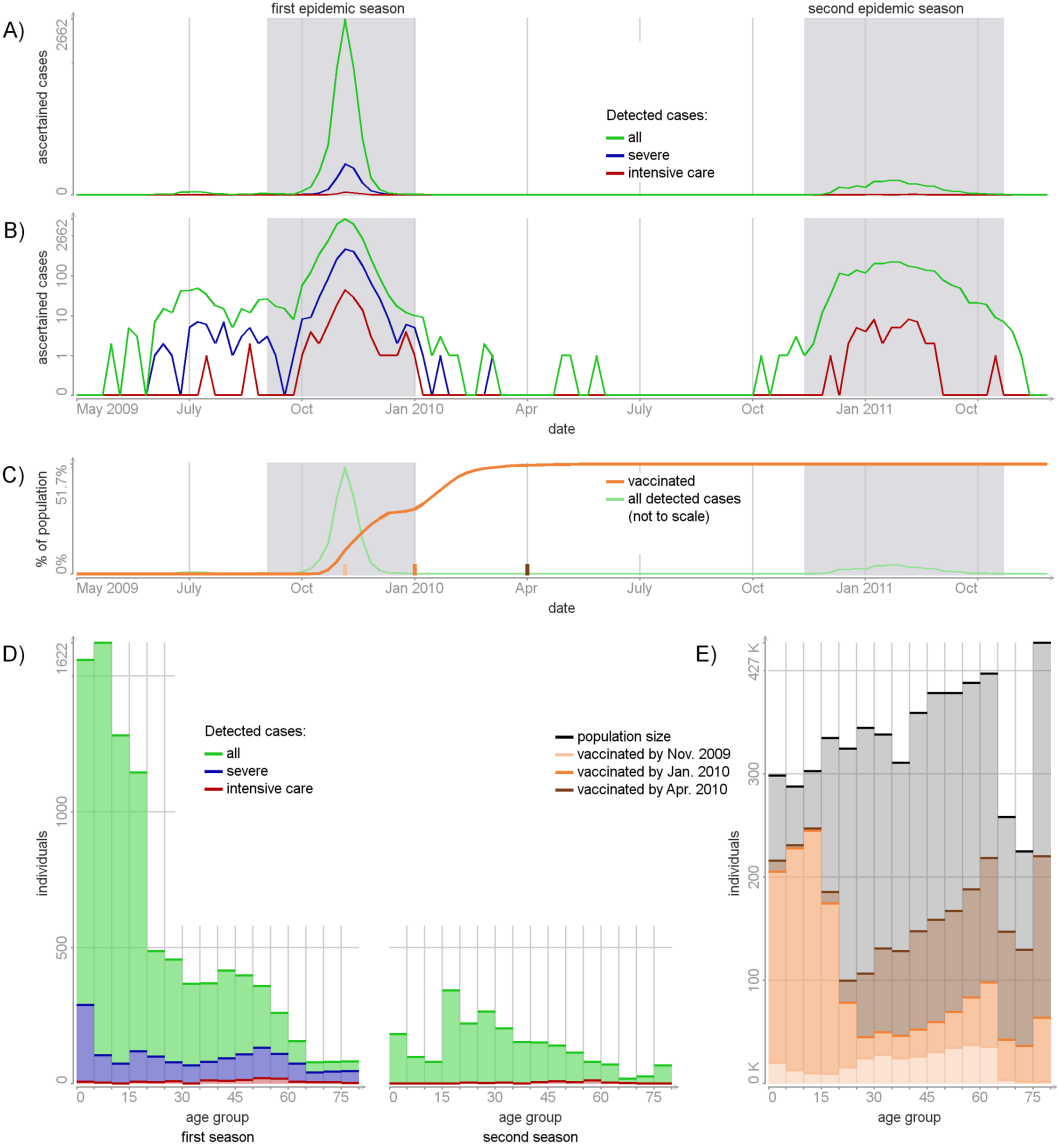
A(H1N1)pdm cases in Finland (2009-2011) and the coverage of vaccination. Panels A and B: the numbers of detected (i.e. registered) cases per week on the absolute and log scales. Panel C: the numbers of individuals vaccinated against A(H1N1)pdm09 per week. The shaded areas mark the first and the second epidemic seasons. Panel D: the numbers of detected cases per age group in the first and second seasons. Panel E: population sizes and the numbers of vaccinated individuals per age group.

In all datasets used in this study, information about individuals was aggregated into 16 age groups: 0-4, 5-9, …, 70-74, 75+ years of age. Fig 1 presents the data on registered A(H1N1)pdm09 cases and the coverage of vaccination in Finland 2009-2011. The population sizes were obtained from Statistic Finland (www.stat.fi).

#### Severe cases

Weekly numbers of severe cases of A(H1N1)pdm09 were obtained from a web-based notification system [1]. For the first season (2009/2010), the data comprise all hospitalized cases, including those that required intensive care (IC) admission and cases with fatal outcome. For the second season (2010/2011), only IC and fatal cases were recorded in this system.

#### Mild cases

Weekly numbers of laboratory-confirmed cases of influenza A in the two seasons were obtained from NIDR (surveillance system of the National Infectious Disease Registry) [1]. Based on the high proportion of the A(H1N1)pdm09 strain among all tested A strains (99% in the first season and 95% in the second season [9]), all A influenza cases were considered as A(H1N1)pdm09 cases. All cases included in NIDR but absent in the web-based system were considered as non-hospitalized and therefore called as mild.

#### Vaccination

The numbers of individuals vaccinated against the A(H1N1)pdm09 strain by week and age group were retrieved from a nation-wide register set up especially for the first season. Before January 2010 the majority of vaccines were distributed among children 0-19 years old. (Fig 1E)

#### Contact rates

The rates of social contacts were estimated from the Finnish arm of the Polymod survey data [10]. The dataset contains information about the daily contacts in a random sample of Finnish residents.

**Fig 2:**
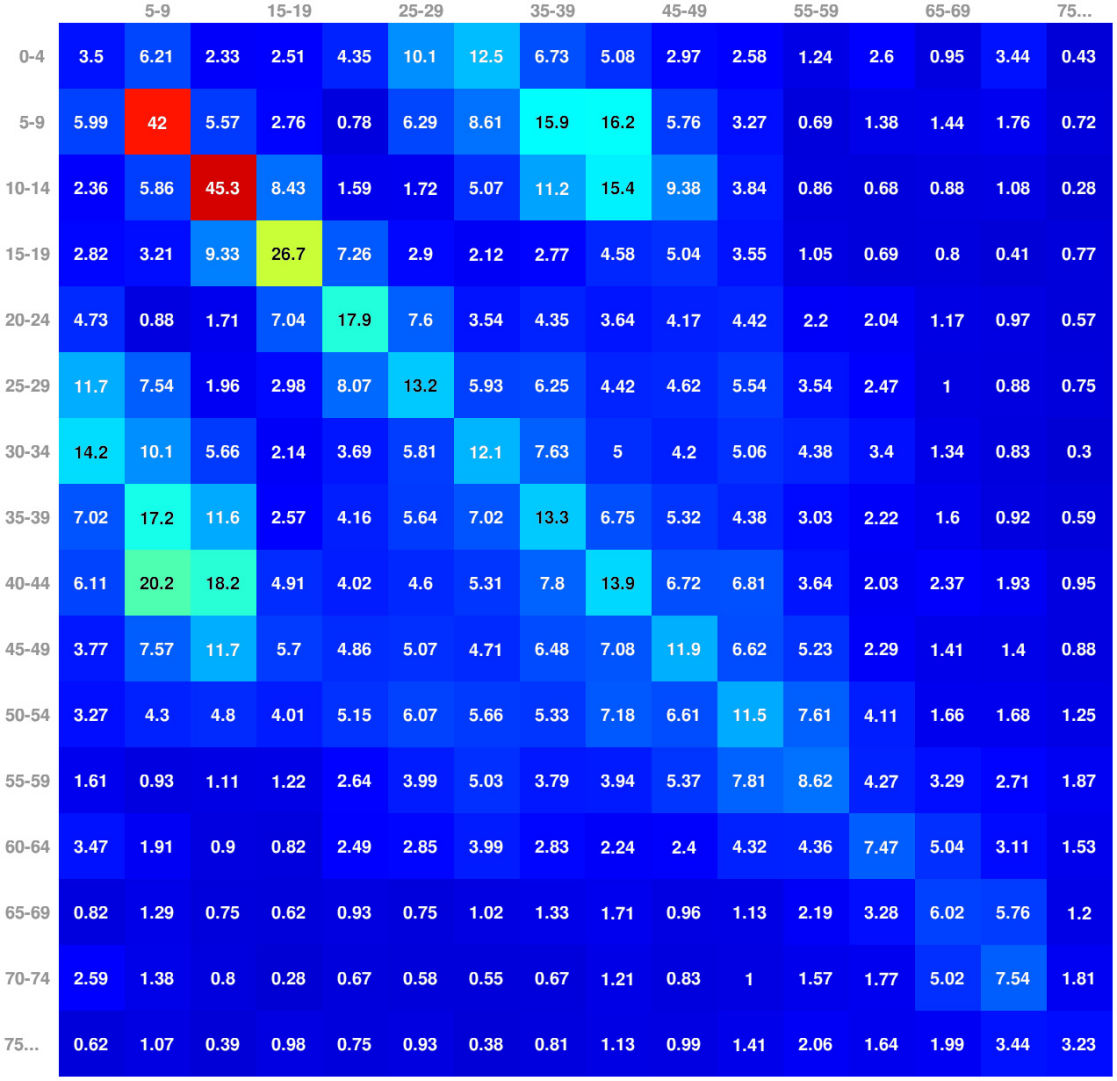
Contact matrix *C*. Each element presents the estimated mean number of weekly social contacts from individual of the column age group to the row age group.

Bayesian modelling was used to estimate a contact matrix *C* with elements *C*_*a*→*b*_ as the mean numbers of potentially infectious contacts produced by a single individual from age group *a* to individuals in age group *b* during one week. The posterior means of *C*_*a*→*b*_ (Fig 2) were used in the further analysis. The estimation of the contact matrix is presented in S1 Appendix. All data is available in S1 Dataset.

### Model of influenza transmission and disease

We built a discrete-time dynamical model of influenza transmission and disease in the Finnish population. The time step was one week, corresponding to the resolution in the data. A period of 113 weeks was modelled from week 15/2009 (one month before the first A(H1N1)pdm09 cases of were registered in Finland) through week 22/2011 (after the end of the second season). Within the modelled period, two subperiods are referred to as the first epidemic season (weeks 37/2009 through 1/2010) and the second epidemic season (weeks 46/2010 through 17/2011).

#### Population structure

**Fig 3:**
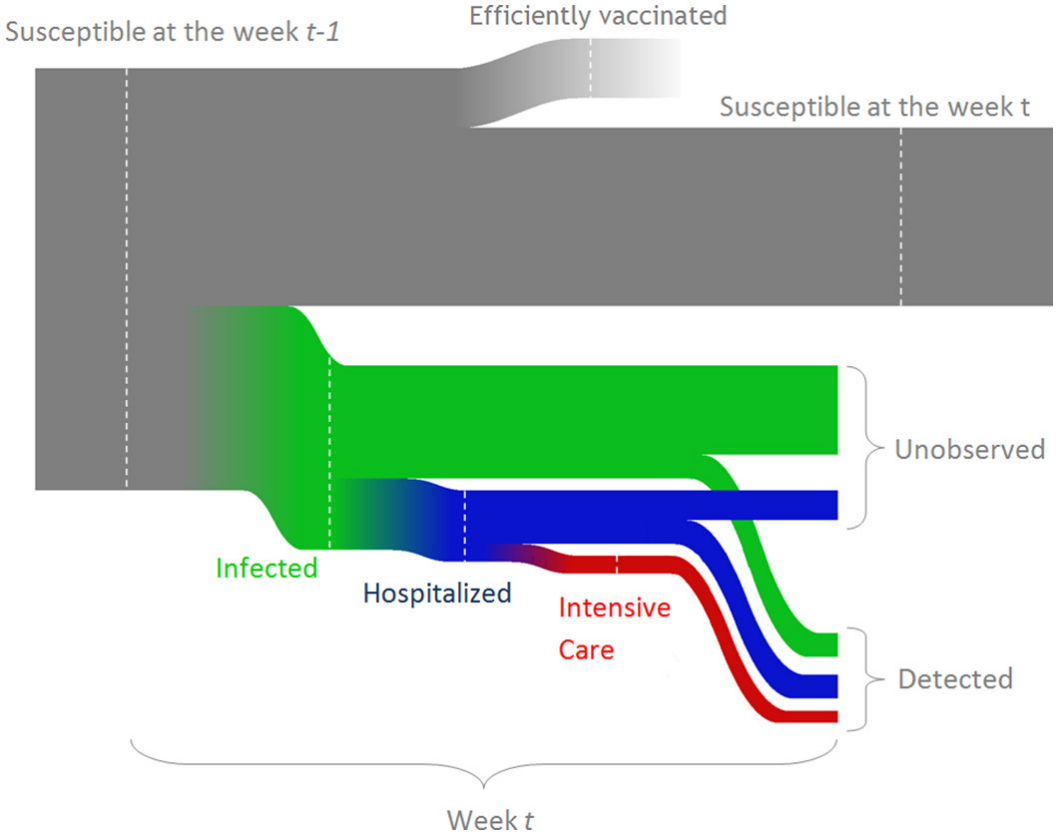
A scheme of possible transitions for an initially susceptible individual per one week. The heights of the lines are indicative of the probabilities of each possible outcome but are not presented at the correct scale.

The model population was divided into *n* = 16 age groups: 0-4, 5-9, …, 70-74, 75+ years of age. Each age group *a* consists of *N*_*a*_ individuals. At each week *t*, there are *S*_*a*,*t*_ susceptible and *I*_*a*,*t*_ infected individuals. We assumed that all infected individual are infectious. At all times, *S*_*a*,*t*_ + *I*_*a*,*t*_ ≤ *N*_*a*_. Fig 3 illustrates the relation between the different subgroups in the population. For simplicity, we assume there are no population dynamics during the modelled period, i.e. no one is born, dies or grows older.

#### Infection and recovery

All individuals are considered to be susceptible at the first week of the modelled period (*S*_*a*,0_ = *N*_*a*_; *I*_*a*,0_ = 0). All individuals that are susceptible at week *t* − 1 are exposed to the infection pressure and can acquire infection at week *t* with probability *r*_*a*,*t*_. Otherwise, they can acquire immunity from vaccination with probability *v*_*a*,*t*_. All individuals, infectious at week *t* − 1 are recovered at week *t*. The recovered individuals are considered to be immune and non-infectious for the rest of the studied period. The values of *I* and *S* for *t* > 0 are distributed according to the following rules:

$$\begin{array}{ccc} I_{a,t} & ∼ & \text{Binom}(S_{a,t-1};r_{a,t}),\\ r_{a,t} & = & 1-\left(1-q_{a}\right){\left(1-\frac{p_{a}}{N_{a}}\right)}^{w_{t}\sum_{b=1}^{16}C_{b\to a}I_{b,t-1}},\\ S_{a,t} & ∼ & \text{Binom}(S_{a,t-1}-I_{a,t};1-v_{a,t}). \end{array}$$

The probability of infection *r*_*a*,*t*_ is expressed as the complement of the probability for an individual to avoid infection from both within and outside the population. Here *q*_*a*_ is the inflow of infection (weekly probability to acquire infection from outside of the population); *p*_*a*_ is the susceptibility (probability that the individual acquires infection per contact with an infectious individual within the population)); $w_{t}\sum_{b=1}^{16}C_{b\to a}I_{b,t-1}$ is the total number of infectious contacts received by age group *a*; *w*_*t*_ is a transmission random effect (autocorrelated multiplicative noise; see section Prior specifications) and *C* is the contact matrix. The expression for *v*_*a*,*t*_ is derived in the next paragraph.

#### Vaccination

We assumed that the A(H1N1)pdm09 vaccine was distributed randomly within each age group, irrespective of the individuals’ current infection status. A vaccinated individual was assumed to develop protective immunity in two weeks with probability 0.8 [11]. Therefore, the probability *v*_*a*,*t*_ of acquiring vaccine-induced protection at week *t* in age group *a* for a still-susceptible individual was calculated as

$$\begin{array}{c} v_{a,t}=0.8\frac{V_{a,t-2}}{N_{a}-\sum_{\tau =0}^{t-3}V_{a,\tau }}, \end{array}$$

where *V*_*a*,*t*_ is the registered number of individuals vaccinated at week *t*.

#### Severity

We assumed three severity classes of A(H1N1)pdm09 infection: (1) mild infection not requiring hospitalization, including asymptomatic cases; (2) infection requiring hospitalization but not admitted to intensive care; and (3) infections admitted to intensive care (IC), including lethal infections; $I_{a,t}=I_{a,t}^{\left(\text{mild}\right)}+I_{a,t}^{\left(\text{hosp}\right)}+I_{a,t}^{\left(\text{IC}\right)}$; the latter two class are referred to as severe infections $I_{a,t}^{\left(\text{severe}\right)}=I_{a,t}^{\left(\text{hosp}\right)}+I_{a,t}^{\left(\text{IC}\right)}$. An infected individual from age group *a* was assumed to develop severe infection with probability $s_{a}^{\left(\text{sev/inf}\right)}$; individual with severe infection was admitted to IC with probability $s_{a}^{\left(\text{IC/sev}\right)}$. The following equations summarise the severity model:

$$\begin{array}{ccc} I_{a,t}^{\left(\text{severe}\right)} & ∼ & \text{Binom}(I_{a,t};s_{a}^{\left(\text{sev/inf}\right)}),\\ I_{a,t}^{\left(\text{IC}\right)} & ∼ & \text{Binom}(I_{a,t}^{\left(\text{severe}\right)};s_{a}^{\left(\text{IC/sev}\right)}). \end{array}$$

#### Detection

Only a portion of infections becomes detected. We assumed that mild infections become detected with a time-varying autocorrelated probability $d_{t}^{\left(\text{mild}\right)}$. Hospitalized non-IC infections are detected with probability *d*^(hosp)^ during the first season (first 60 weeks) and 0 otherwise (as no hospitalized non-IC cases were recorded during the second season). IC infections are always detected. Let $D_{a,t}^{\text{(severity}\;\text{class)}}$ denote the number of cases in the severity class in age group *a* at week *t* registered in the data. The following equations summarise the detection model:

$$\begin{array}{ccc} D_{a,t}^{\left(\text{mild}\right)} & ∼ & \text{Binom}(I_{a,t}^{\left(\text{mild}\right)};d_{t}^{\left(\text{mild}\right)}),\\ D_{a,t}^{\left(\text{hosp}\right)} & ∼ & \text{Binom}(I_{a,t}^{\left(\text{hosp}\right)};d^{\left(\text{hosp}\right)}1\left(t<60\right)),\\ D_{a,t}^{\left(\text{IC}\right)} & = & I_{a,t}^{\left(\text{IC}\right)}. \end{array}$$

#### Simplifying assumptions

To improve the identifiability of the model parameters we made the following simplifying assumptions. The four parameters (*p*, *q*, *s*^(sev/inf)^, *s*^(IC/sev)^) were considered in 6 wider age strata: 0-4, 5-14, 15-19, 20-29, 30-64, 65+ years. We set *p*_*a*_ = *p*_*b*_, *q*_*a*_ = *q*_*b*_, $s_{a}^{\left(\text{sev/inf}\right)}=s_{b}^{\left(\text{sev/inf}\right)}$ and $s_{a}^{\left(\text{IC/sev}\right)}=s_{b}^{\left(\text{IC/sev}\right)}$ if age groups *a* and *b* belong to the same stratum. The infection model still has 16 age groups.

#### Reproduction numbers

The number of secondary infections produced by a single infected individual in a totally susceptible population (basic reproduction number *R*_0_) in a discrete-time model could be calculated as the largest eigenvalue of the next generation matrix [12], the elements of which in our discrete-time model are approximated by *NGM*_*a*,*b*_ ≈ *p*_*a*_ *C*_*b*→*a*_ (see S2 Appendix). In the presented model, the contact matrix is additionally multiplied by the time-dependent random effect *w*_*t*_ with prior mean 1. The basic reproduction number at any particular week *t* was therefore calculated as *R*_0,*t*_ = *w*_*t*_ *R*_0_.

The number of secondary cases produced by a single infected individual in age group *a* in a totally susceptible population can be approximated by $R_{a}=\sum_{b=1}^{16}p_{b}C_{a\to b}$. The effect of the inflow of the infection is measured as the mean number of infections induced to a totally susceptible population during one week from the outside and is equal to *N*_*a*_ *q*_*a*_.

**Fig 4:**
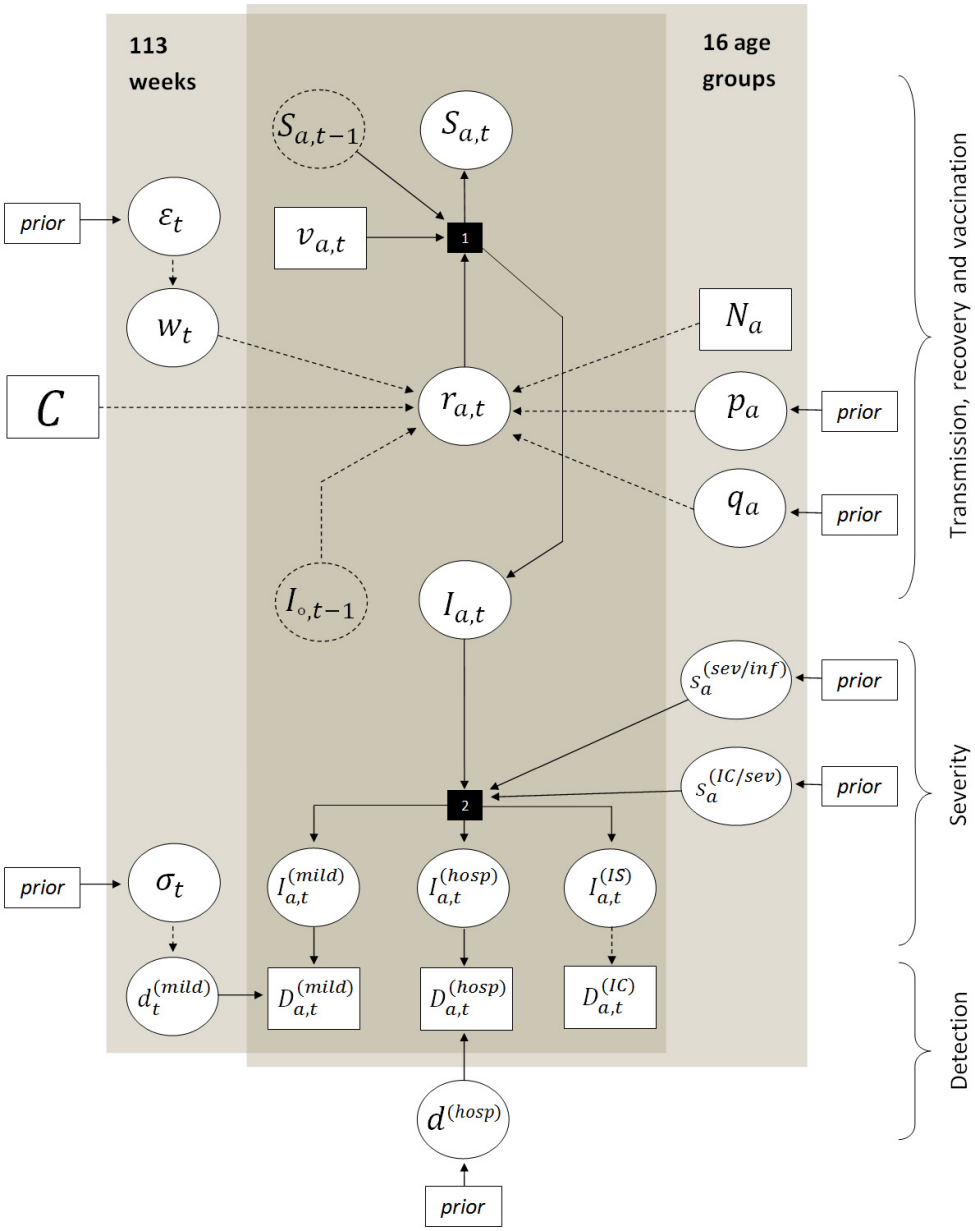
DAG of the model. Circles represent model unknowns, rectangles known or fixed values. The plates highlight the values specified for each week and/or age group. Dotted circles are used to show the relations between strata. Smaller rectangles with “prior” sign point out those model parameters with specified prior distributions. Stochastic relations are indicated with solid lines, deterministic with dashed lines. Complex relations are shown as black rectangles: 1—infection process, 2—detection process.

Table 1 summarizes the unknown model parameters, while Table 2 summarizes all estimated quantities. The combined model equations as well as the model DAG (directed acyclic graph) are shown in Fig 4.

**Table 1:** Model parameters. The parameters are divided under four topics.

Topic	Parameter	Meaning	Prior	Source
Susceptibility:	*p*_*a*_	Probability for a susceptible in age group *a* to acquire infection per contact with an infectious host within the population	Uniform(0, 1)	Uninformative
	*q*_*a*_	Inflow of infection (probability to acquire infection from outside the population per week) in age group *a*	Beta(0.1, 1)	Assumed to be small
Transmission:	*w*_*t*_	Transmission random effect at week *t*	2 LogitNormal(0.5, 1)*	Uninformative
Severity:	$s_{a}^{\left(\text{sev/inf}\right)}$	Hospitalization/infection ratio in age group *a*	LogitNormal(0.01, 0.1)	[13]
	$s_{a}^{\left(\text{IC/sev}\right)}$	IC/hospitalization ratio in age group *a*	LogitNormal(0.1, 0.1)	[13]
Detection:	$d_{t}^{\left(\text{mild}\right)}$	Mild case detection probability at week *t*	LogitNormal(0.01, 0.01)*	[14][15][16]
	*d*^(hosp)^	Hospitalized non-IC case detection probability	LogitNormal(0.75, 0.1)	Expert opinion

Here the LogitNormal(*x*, *y*) means a distribution of a random variable for which the logit transformation has a normal distibution with mean *logit*(*x*) and variance *y*.

* An autocorrelated prior is constructed for both *w*_*t*_ and $d_{t}^{\left(\text{mild}\right)}$; see Prior specifications

**Table 2:** Latent variables and estimated quantities.

Topic	Quantity	Meaning
Incidence:	*I*_*a*,*t*_	True number of infections in age group *a* at week *t*
	∑_*t*∈*T*_ *I*_*a*,*t*_/*N*_*a*_	Attack rate during period *T* in age group *a*
Transmission:	*R*_0,*t*_ = *R*_0_ *w*_*t*_	Basic reproduction number at week *t*
	*R*_*a*_ = ∑_*b*_ *p*_*b*_ *C*_*a*→*b*_	Reproduction number for age group *a*
	*q*_*a*_ *N*_*a*_	Effect of the inflow
Detection:	∑_*a*,*t*∈*T*_ *D*_*a*,*t*_/∑_*a*,*t*∈*T*_ *I*_*a*,*t*_	Detection ratio during period *T*

### Prior specifications

The prior distributions of the model parameters are presented in Table 1. All parameters except the age-dependent susceptibility *p* and transmission random effect *w*_*t*_ were given informative priors. The severity parameters *s*^(sev/inf)^ and *s*^(IC/sev)^ were centred around 1% and 10%, respectively. The detection probabilities $d_{t}^{\left(\text{mild}\right)}$ and *d*^(hosp)^ were centred around 1% and 75%, respectively. These priors were consistent with the ones used in our earlier analysis of the same data [2]. The inflow of infection *q* was assumed to be extremely small.

#### Prior for the temporal correlation

The time-dependent transmission random effect *w*_*t*_ represents a combination of time-dependent factors affecting the spread of infection but not accounted for by the transmission model (e.g. air temperature, humidity, population response to the epidemic, public holidays). The time-dependent mild case detection probability $d_{t}^{\left(\text{mild}\right)}$ represents a combination of factors affecting the detection of mild cases (e.g. the changes in reporting policies for A(H1N1)pdm09 in Finland, changes in public concerns about the epidemic which could affect the individual’s willingness to seek medical care).

*A priori* we believe that parameters *w*_*t*_ and $d_{t}^{\left(\text{mild}\right)}$ are autocorrelated (but independent of each other). To represent this, we construct them as realisations of logistic-transformed multivariate *T*-dimensional (*T* = 113) normal random variables *ε* and *σ*, with the covariance defined by kernel *K*:

$$\begin{array}{ccc} w_{t} & = & 2\;\text{logistic}(\varepsilon_{t}),\\ d_{t}^{\left(\text{mild}\right)} & = & \text{logistic}(\sqrt{0.01}\;\sigma_{t}+\text{logit}\left(0.01\right))\;\text{for}\;t\in 0\cdots T-1,\\ \varepsilon ,\sigma & ∼ & N(\underset{T}{\underset{︸}{\left(0,0\cdots 0\right)}},K),\\ K_{i,j} & = & \exp{\left(-(i-j\right)}^{2}/52)+0.01\times 1\left(i=j\right)\;\text{for}\;i,j\in 0\cdots T-1. \end{array}$$

Here *T* = 113 is number of weeks in the modelled period and 1(i=j) is 1 if *i* = *j* and 0 otherwise. The marginal prior distribution of *ε*_*t*_ and *σ*_*t*_ is N(0,1.01), therefore the marginal prior distribution of *w*_*t*_ is concentrated around 1 and distributed in the interval [0, 2] while the prior of $d_{t}^{\left(\text{mild}\right)}$ is concentrated around 0.01 for every *t* and distributed in the interval [0, 1] (see Table 1).

The prior values of *ε*_*t*_ correlate within few months. For example, the prior correlation between *ε*_*i*_ and *ε*_*j*_ is 0.98 for |*i* − *j*| = 1 week, 0.5 for |*i* − *j*| = 6 weeks, 0.06 for |*i* − *j*| = 12 weeks. The same applies for *σ*.

### Computational method

We estimated the joint posterior distribution of the model parameters and latent variables using Markov chain Monte Carlo computation (MCMC) with particle Gibbs sampler step [17]. In addition, we applied exact approximate MCMC [18] targeting a smoothed marginal posterior of the model parameters, *p*(*parameters*|*data*)^1/25^ and *p*(*parameters*|*data*)^1/5^, to ensure that the peak area of the target posterior is unimodal and well-behaving. Details are provided in S3 Appendix.

Posterior predictive checks were used to to explore how well the model describes the observed data. Sensitivity analysis was performed by comparing the posterior modes (i.e. maximum *a posteriori* estimates) under different prior settings. Details are provided in S5 Appendix.

## Results

If not otherwise stated, the results will be presented in terms of 90% posterior intervals (i.e. the 5^th^ and 95^th^ percentiles) of the estimated quantities. Additional results are presented in S4 Appendix. Exact numerical estimates are presented in S2 Dataset.

### Incidence

**Fig 5:**
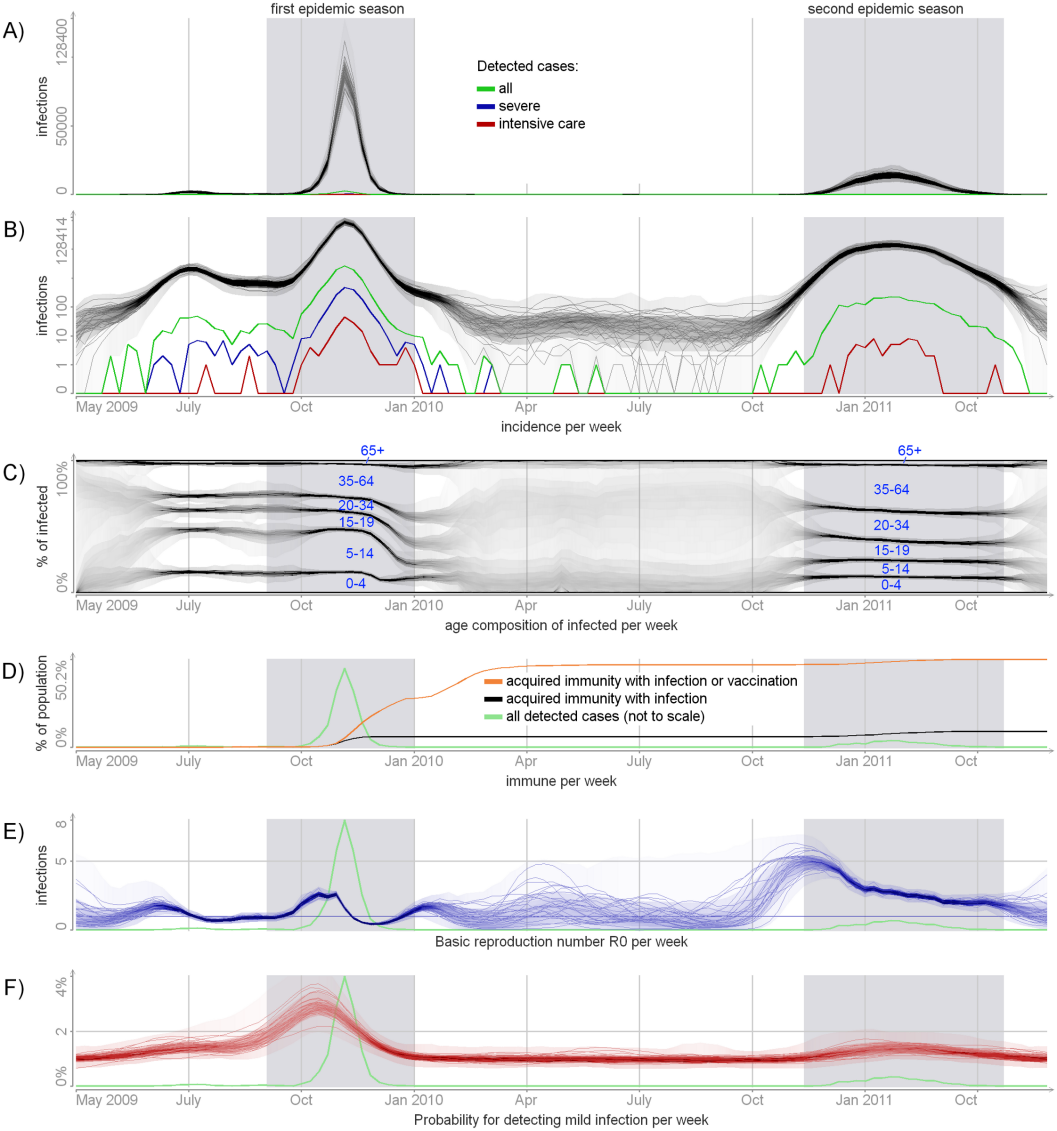
Posterior distribution of time-dependent unknowns. Panels A and B: the true incidence in absolute and log scales. The detected numbers are shown for reference. Panel C: the cumulative age distribution of infected individuals. Panel D: the estimated number of immune individuals per week. Panel E: the basic reproduction number *R*_0,*t*_ = *R*_0_ *w*_*t*_ per week. Panel F: the probability of detecting mild infection per week $d_{t}^{\left(\text{mild}\right)}$. The full posterior distributions are visualized, with more probable values represented by darker color. In addition, a few samples from the posterior are shown. The shaded areas mark the epidemic seasons.

**Fig 6:**
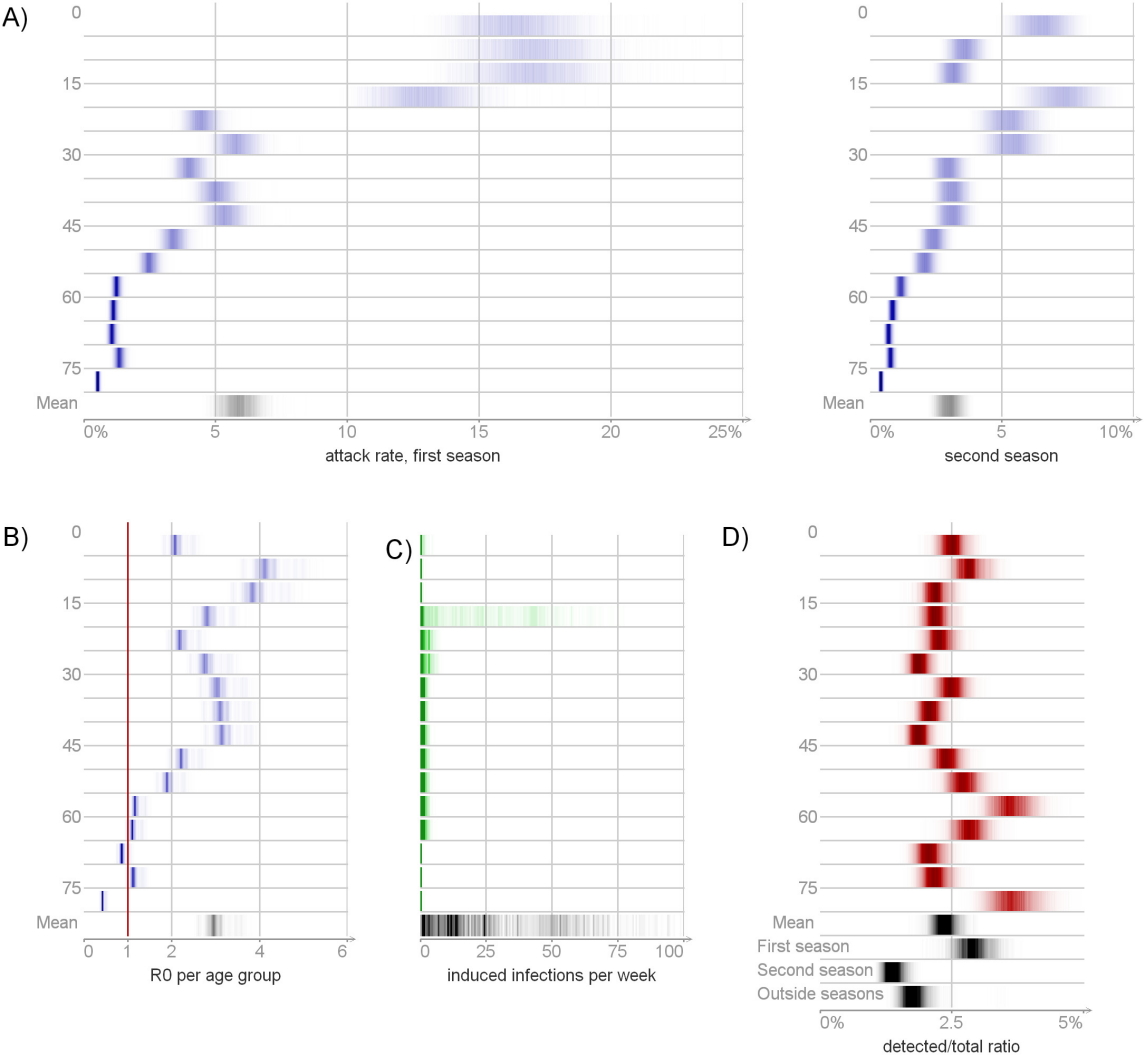
Posterior distribution of derived quantities (Table 2). Panel A: the attack rates per season and age group. Panel B: basic reproduction numbers *R*_0_. Panel C: the effect of the inflow of infection per age group. Panel D: detection ratios. The full posterior distributions are visualized, with more probable values represented by darker color.

The estimated true numbers of A(H1N1)pdm09 infection are shown in Fig 5A and 5B. Fig 6A presents the attack rates, i.e. the numbers of infected per population size. We estimated that 440 000 – 550 000 individuals in total (8.2 – 10.4% of the population, posterior mean 500 000, 9.3%) were infected in Finland during the modelled period. Specifically, the numbers infected were 270 000 – 360 000 (5.1 – 6.7%, posterior mean 320 000, 5.9%) and 140 000 – 190 000 (2.5 – 3.5%, posterior mean 160 000, 3.0%) during the first and the second A(H1N1)pdm09 epidemic seasons, respectively. Only a minor portion of infections (0.3 – 0.4%) occurred outside the two epidemic seasons. In both seasons, the attack rate decreased with age. It was largest in the youngest age group (14 – 19% during the first and 5.5 – 7.6% during the second epidemic season) and smallest in the oldest (5.0 – 6.6% and 4.6 – 6.4%).

Fig 5C presents the cumulative age composition of the infected population per week. The mean age of infection increased with time. Before the peak of the first season, approximately half of all infections occurred among less then 15 years olds. During the second epidemic season only 25% of infections belonged to this age group. The oldest (65+ years) never accounted for a significant portion of the infected population.

### Susceptibility

**Fig 7:**
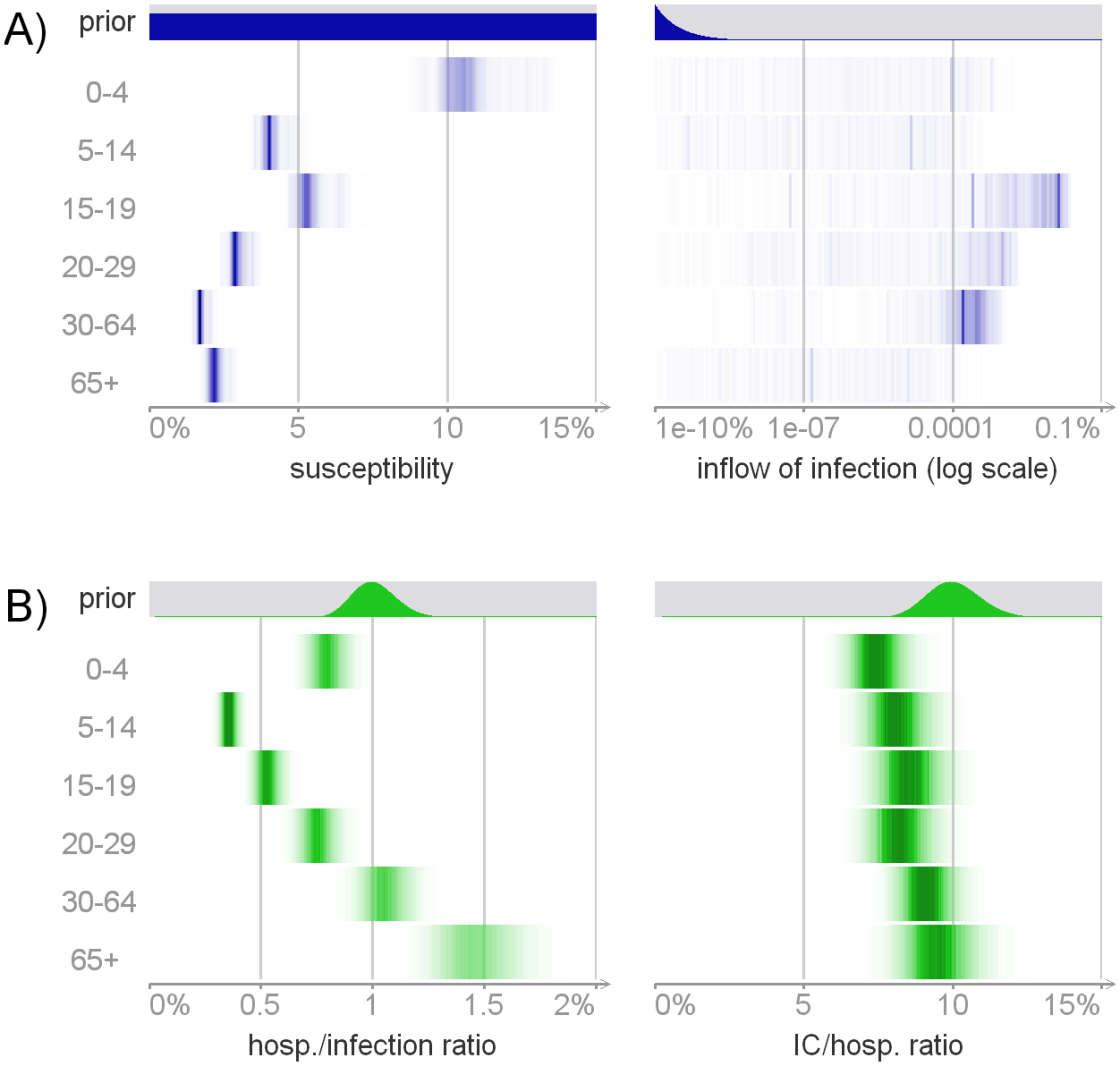
Posterior distributions of model parameters (Table 1). Panel A: the susceptibility *p* and the inflow of infection *q*. Panel B: severities *s*^(sev/inf)^ and *s*^(IC/sev)^. The full posterior distributions are visualized, with more probable values represented by darker color. The prior distributions of these parameters are shown for reference.

Fig 7A shows the posterior distribution of susceptibility *p* (probability of acquiring infection per contact with an infectious individual) and inflow *q* (probability of acquiring infection from outside the population). Susceptibility decreased with age: children aged less than 5 years had a 5-fold greater chance to acquire infection per contact than the oldest individuals. Individuals aged 20-29 years were most likely to acquire infection from outside the population.

### Severity

Fig 7B shows the posterior distributions of the severity parameters *s*^(sev/inf)^ and *s*^(IC/sev)^. The hospitalization/infection ratio had a V shape, the infection being more severe among the youngest (*s*^(sev/inf)^ = 0.7 – 0.9%) and the oldest (1.3 – 1.7%). Children aged 5-14 years had the smallest probability of severe disease per infection (0.3 – 0.4%). The IC/hospitalization ratio did not vary much across age groups, almost repeating the prior information. It was smallest among the youngest (*s*^(IC/sev)^ = 7 – 8%) and largest (8 – 11%) for those over 30 years.

### Transmission and seasonality

In our model, influenza transmission, including the outbreaks and periods between epidemic seasons, is modulated by a time-varying reproduction number *R*_0,*t*_ = *R*_0_ *w*_*t*_ (Fig 5E). Before June 2009, *R*_0,*t*_ rose above 1 allowing for the minor pre-seasonal outbreak. A significant increase in *R*_0,*t*_ in the autumn of 2009 marked the onset of the first epidemic season. After the peak of the first season (November 2009) *R*_0,*t*_ dropped below 1 leading to the end of the first outbreak.

By the end of the first epidemic season about 22% of the population were vaccine-protected (Fig 5D), especially in the youngest age groups (53% in <20 year olds, 14% in 20-64 olds, and 11% in >65 olds). This induced herd immunity in the population, so *R*_0,*t*_ could raise above 1 without causing an outbreak. By the second epidemic season, 41% of the population acquired immunity from vaccination (posterior mean 52%, 34%, and 47% of individuals aged 0-19, 20-64 and older than 64 years, respectively) and 5 – 7% acquired immunity from infection.

Around October 2010 *R*_0,*t*_ started gradually increasing, reaching its maximum in November 2010 and then slowly decreased. For the period November 2010—January 2011, the reproduction number was above 3. This marked the second epidemic season.

The estimates of *R*_0,*t*_ outside the epidemic seasons are uncertain, as scarce data are available for these periods. Overall, the product *R*_0,*t*_ = *R*_0_*w*_*t*_ was estimated with smaller uncertainty than *w*_*t*_ and *R*_0_ individually (see S4 Appendix).

The largest number of potential infections was produced by individuals from age groups 5-14 years old (3.5 – 5.6 infections) (Fig 6B). The smallest number was produced by the oldest age group (0.4 – 0.5 infections). On average, only few infections per week were introduced from outside the population (Fig 6C). The random effect *w*_*t*_ and the detection probability $d_{t}^{\left(\text{mild}\right)}$ increased simultaneously during the early phases of the epidemic seasons. However, for any time (*t* ∈ 0, …, *T* − 1), the variables *w*_*t*_ and $d_{t}^{\left(\text{mild}\right)}$ did not have strong posterior correlation (see S4 Appendix).

### Detection

Fig 6D shows the number of detected cases per the number of infected (detection ratio; Table 2). We estimated that 2.1 – 2.7% of all A(H1N1)pdm09 infections were detected (specifically 2.5 – 3.3% during the first epidemic season, 1.2 – 1.6% during the second and 1.5 – 2.0% outside seasons). The detection ratio varied by age with posterior means ranging from 3.7% to 1.9%.

We estimated that the detection probability of the mild cases $d_{t}^{\left(\text{mild}\right)}$ reached its maximum before the peak of the first season and decreased subsequently during the outbreak (Fig 5F). During November 2009, the observed numbers of mild infections decreased much faster than the observed numbers of hospitalized cases. According to the model, however, the true numbers of mild and severe infections decreased at the same speed and the observed difference was thus explained by the decline in $d_{t}^{\left(\text{mild}\right)}$. The posterior of the detection probability of hospitalized cases *d*^(hosp)^ followed the prior closely.

### Effect of the vaccination campaign

**Fig 8:**
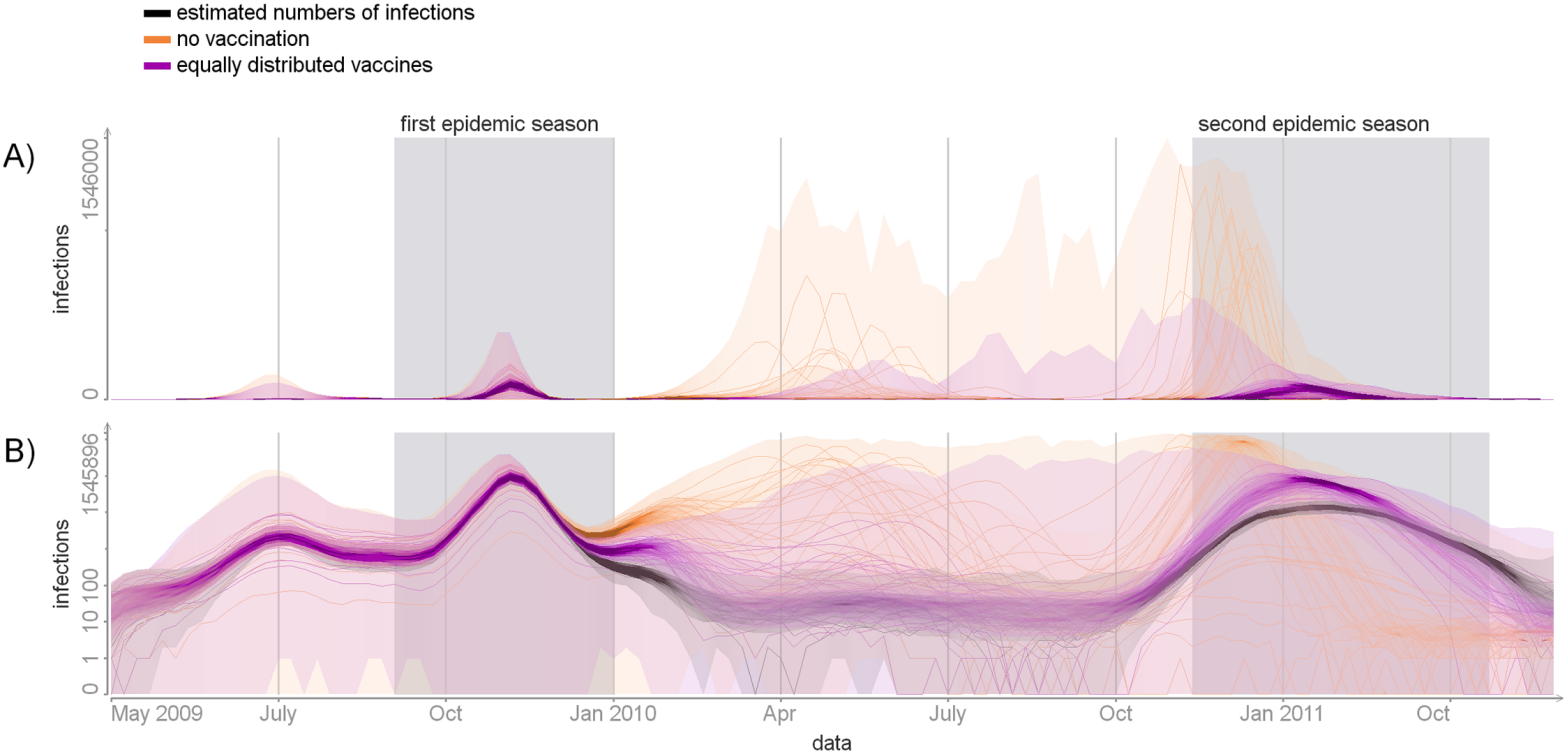
Simulated scenarios. The numbers of infections per week in simulations in absolute (panel A) and log (panel B) scales. Orange colour—scenario with no vaccination. Magenta colour—scenario with vaccines equally distributed among age groups. The posterior distribution of incidence is shown in black for reference. The full probability distributions are visualized, with more probable values represented by more concentrated color. In addition, a few samples are shown.

We measured the impact of the vaccination campaign as the number of cases prevented. To estimate this number, we simulated the incidence of infection, using parameter values sampled from the posterior and assuming that no one was vaccinated (*v*_*a*,*t*_ = 0). According to this analysis (Fig 8), the second season could have started earlier and caused a larger outbreak, leading to 4-8 times more infections overall (total attack rate would have been 38 – 78%). By contrast, vaccination did not affect the first epidemic season.

We also estimated the impact of the vaccination under a scenario where vaccines were distributed in the same amount but independent of age (*v*_*a*,*t*_ = *v*_*b*,*t*_ for all age groups *a*, *b*). In this situation our model predicts about twice as many infections overall (total attack rate would have been 15 – 26%).

## Discussion

Using a dynamic transmission model, we estimated that 5.9% (90% credible interval 5.1 – 6.7%) of the Finnish population was infected during the first year of the pandemic A(H1N1)pdm09 strain of influenza in 2009/2010. There was a second season a year later with an attack rate of 3.0% (2.5 – 3.5%) of the population. The vaccination campaign launched in the middle of the first epidemic epidemic season was essential in mitigating the size of the second season, but occurred too late to have an impact on the first season. In both seasons, the proportion of the infected population decreased with age, with the youngest being at least an order of magnitude more likely to be infected than the oldest.

The age distribution of the infected population evolved over time. Before the peak of the first season most infected individuals were children aged less than 15 years. According to the social mixing matrix, estimated from the available data, this age group forms the core group of transmission for infections that spread through droplets in close contact. After the end of the first season, as many as 72% of children aged less than 15 years either had had natural infection (18%) or had been vaccinated (55%) so that the importance of this age group in the chain of transmission decreased. During the second epidemic season, the mean age of infected individuals was higher.

The posterior mean severity of influenza infection, as measured by the hospitalization/infection ratio (parameter *s*^(sev/inf)^), was 0.7% when averaged over all age groups and had a clear V shape with the youngest and oldest requiring hospitalization more often. The IC/hospitalization ratio (parameter *s*^(IC/sev)^) was driven almost entirely by prior information (around 8% across all age groups).

We estimated that only 2.4% (90% credible interval 2.1 – 2.7%) of infections were recorded by surveillance, i.e. there were 40 – 50 unobserved A(H1N1)pdm09 infections for each detected case. The detection probability peaked early during the first epidemic season, with a clear decline towards the end of the season (Fig 5F). This could reflect the public and governmental concerns increasing initially and then declining as the awareness of the relatively mild impact of the novel A(H1N1)pdm09 virus was revealed. The detection ratio in the second epidemic season was smaller than in the first one. Similar patterns in the detection rates occurred in the UK during the first two years of the pandemic [19].

In our model, the spread of infection is modulated by four quantities: susceptibility to infection (parameter *p*), the pattern of contacts (contact matrix *C*), the time varying reproduction number (*R*_0,*t*_) and the rate of inflow of infection (*q*). Susceptibility to infection was estimated to decrease with age, which is likely to reflect higher levels of pre-existing immunity among older individuals [7]. The contact matrix was based on a survey of daily social contacts in Finland [10].

The standard deterministic SIR model assumes that outbreaks only stop by depletion of the pool of susceptibles. In particular, a second season would be impossible unless the virus evolves to escape the prevailing immunity in the population. Although this is known to happen for seasonal influenza [20], the virus did not change much during the first two years of the pandemic [7]. Vaccination alone cannot explain the observations, as the first season ended in the population with 20% vaccine-induced protection while the second season started with 40%. Therefore, stochasticity in transmission and other mechanisms may be called for.

We applied a time-varying reproduction number (*R*_0,*t*_) of influenza transmission, capturing the impact of changing population behaviour or weather conditions as a stochastic process. In particular, cold and dry weather has been suggested as one of the drivers of influenza transmission [21] and the public behaviour may have changed as the epidemic appeared to be relatively mild. We estimated that *R*_0,*t*_ changed markedly with time (Fig 5E).

The model explains the appearance of the second epidemic season when almost half of the population was immune with extraordinary transmission circumstances: the reproduction number was very large (*R*_0,*t*_ > 3) for a period October 2010 through January 2011, possibly reflecting a seasonal (weather) effect. Dorigatti et al. [19] reported a similar finding regarding the 3rd wave of A(H1N1)pdm09 in the UK, one year after the 1st and 2nd waves. They inferred that the basic reproduction number increased to 1.5 before the 3rd wave and concluded that this was likely due to the combination of favourable weather conditions and possible evolution of the virus. Of note, we did not factor the possibility of waning immunity in the analysis and, should it have occurred, our estimate of *R*_0,*t*_ would be too high.

The rate of introduction of infection to the population (parameter *q*) mainly plays the role of a primer that initiates the outbreaks. Its influence during the outbreaks (epidemic seasons) was insignificant. Its role was to add stochasticity to the onsets of influenza seasons, thus removing the need to introduce index cases at any fixed time. The estimates of *q* were notable only for age group 15-29 years, reflecting the fact that the first detected cases in the country belonged to these age groups.

According to our analysis, vaccination played an important role in mitigating A(H1N1)pdm09 transmission in the second season. By the start of the second season, 41% of the population were vaccine-protected while less than 5 – 7% had acquired immunity from infection. We estimated that in the absence of vaccination the affected population would have been about 4 – 8 times larger. It should be noted, however, that these predictions rely heavily on the posterior estimate of the transmission random effect (*w*_*t*_), which in turn may be strongly dependent on the conditions and data in the 2010/2011 season.

In a previous analysis of the same data set [2], we assumed that vaccination did not affect the first season at all, which agrees with the current estimates. Given the two weeks period needed to develop protective immunity after vaccination, it is likely that an effective proportion of immune individuals was only obtained after the end the first epidemic outbreak (Fig 5D). In this study we modelled the vaccine as having a 80% chance to induce complete immunity against the infection. This efficacy was based on a cohort study conducted in a sub-sample of the same population during the same time (i.e. the first season of A(N1H1)pdm09) [11].

We used a discrete-time SIR model with a one-week time step to correspond to the available data. However, a shorter time step would have been more realistic for capturing the dynamics of influenza for which the infectious period is known to last less than a week [6]. In this case, each infection generation in our model likely reflects several actual generations, therefore the basic reproduction number *R*_0,*t*_ is an overestimate of what would have been obtained with a smaller time step or a continuous model.

The estimability of model parameters was constrained by the amount of available data. We used informative priors on all of the model parameters except the susceptibility *p* and the transmission random effect *w*_*t*_ and set a strong smoothness constraint on the time-varying processes of transmission (*w*_*t*_) and detection ($d_{t}^{\left(\text{mild}\right)}$). We conducted several sensitivity analyses to study the impact of the choice of the prior distributions (S5 Appendix). We found that increasing the variance of the prior distributions leads to smaller attack rates and vice versa. The prior of the detection probability for mild cases $d_{t}^{\left(\text{mild}\right)}$ was the most influential one. Some estimated quantities were more robust to prior specification. The detection probability $d_{t}^{mild}$ was always estimated to increase before the outbreak of the first season. The estimated trends, e.g. the decreasing susceptibility with age and the V shape in the age-specific severity, were also immune to the choice of the prior. The reproduction number *R*_0,*t*_ always increased before the seasonal outbreaks.

In our previous study [2] we analysed the same period of the two first years of pandemic influenza in Finland using a static model. A dynamic approach allowed us to take into account the available time-series data to learn about trends in transmissibility and detection. The presented model estimated the total incidence to be 1.5 times higher (see Table 3). A dynamic model also produced larger estimates of the impact of vaccination as it is able to take into account herd immunity effects. Using a static model, we estimated previously that without vaccination the overall attack rate would increase only by 0.8 percentage points.

**Table 3:** Estimates of the attack rates and severity of the pandemic A(H1N1)pdm09 influenza in different regions.

Region and time	Data type	Model	Attack rate	Severity
Finland 2009/2010 and 2010/2011 (the current study)	Laboratory-based surveillance of cases over time; coverage of vaccination over time	Dynamic	5.9% and 3.0% during the two seasons (17% and 3.5% in age group 10-14 years)	Hospitalization/infection ratio 0.7% (0.4% in age group 5-14 years); intensive care/hospitalization ratio 8%
Finland 2009/2010 and 2010/2011 (the same data as in the current study) [2]	Laboratory-based attack rates per season; coverage of vaccination	Static	3.9% and 1% during the two seasons (11% and 2.4% in age group 10-14 years)	Hospitalization/infection ratio 1.1% (0.3% in age group 5-14 years); intensive care/hospitalization ratio 10%
London, two outbreaks, August 2009 and Sep-Dec 2009 [6]	Laboratory-based surveillance of cases over time; incidence of influenza-like illness over time; seroconversion rates	Dynamic	9% and 10% during the outbreaks (22% and 30% in age group 5-14 years)	Not estimated
Several regions, 2009/2010 [3]	Pre- and post-pandemic sera	Static	24% in 2009/2010 (meta-analysis); 46% in age group 5-19 years	Symptomatic disease/infection ratio 1/3, fatal cases/infection ratio 0.02%
UK, three waves: summer 2009, autumn and winter 2009/2010, autumn and winter 2010/2011 [4]	Laboratory-based surveillance of cases per wave; incidence of influenza-like illness; serological surveys	Static	5%, 10% and 15% in three waves. 10%, 20% and 10% in age group 5-14 years	Hospitalization/infection ratio 0.2%; intensive care/infection ratio 0.03%
Netherlands, a single season in autumn-winter 2009 [5]	Laboratory-based surveillance; serological surveys pre- and post season 2009/2010	Static	8%, with 35% in age group 5-19	Hospitalization/infection ratio 0.14%; intensive care/infection ratio 0.017%

The attack rate refers to the (estimated) proportion of infections occurring during one epidemic season. Definitions of severity vary according to study, based on different types of data. For convenience, the estimates from the current study are shown on the first row.

The attack rates and severity of A(H1N1)pdm09 varied considerably by geographical region (see Table 3). Such variation may be partly due to lack of precision, based on the differences in data availability and in the methods of analysis. Nevertheless, the estimated attack rate in Finland was still smaller than found in other studies. Because of the high per-population risk of hospitalization in Finland (0.06%), the severity of infection (hospitalization/infection ratio) was higher in Finland than elsewhere, probably reflecting differences in the health care system and surveillance. Such differences emphasise the need to calibrate transmission models in each particular setting to best address questions about the performance of surveillance and the impact of influenza seasons.

## Supporting Information

S1 Appendix Contact rates. (PDF)

S2 Appendix The model of influenza transmission and disease. (PDF)

S3 Appendix Computational methods. (PDF)

S4 Appendix Additional results. (PDF)

S5 Appendix Additional analyses. (PDF)

S6 Appendix Continuous and discrete-time SIR models. (PDF)

S1 Dataset Numbers of detected cases, vaccinated individuals, population sizes and contact rates. (TXT)

S2 Dataset Estimated parameters and hidden states. (TXT)

## References

[1] Lyytikäinen O, Kuusi M, Snellman M, Virtanen M, Eskola J, Ronkko E, et al Surveillance of influenza in Finland during the 2009 pandemic, 10 May 2009 to 8 March 2010. Euro Surveill. 2011;16(27). 21794216

[2] Shubin M, Virtanen M, Toikkanen S, Lyytikainen O, Auranen K. Estimating the burden of A(H1N1)pdm09 influenza in Finland during two seasons. Epidemiol Infect. 2014 5;142(5):964–974. 10.1017/S0950268813002537 24139316 PMC4097990

[3] Van Kerkhove MD, Hirve S, Koukounari A, Mounts AW, Allwinn R, Bandaranayake D, et al. Estimating age-specific cumulative incidence for the 2009 influenza pandemic: a meta-analysis of A(H1N1)pdm09 serological studies from 19 countries. Influenza Other Respi Viruses. 2013 Jan; Available from: 10.1111/irv.12074 PMC5781221 23331969

[4] Presanis AM, Pebody RG, Birrell PJ, Tom BDM, Green HK, Durnall H, et al Synthesising evidence to estimate pandemic (2009) A/H1N1 influenza severity in 2009-2011. Ann Appl Stat. 2014 12;8(4):2378–2403. Available from: 10.1214/14-AOAS775 10.1214/14-AOAS775

[5] Steens A, Waaijenborg S, Teunis PF, Reimerink JH, Meijer A, van der Lubben M, et al Age-dependent patterns of infection and severity explaining the low impact of 2009 influenza A (H1N1): evidence from serial serologic surveys in the Netherlands. Am J Epidemiol. 2011 12;174(11):1307–1315. 10.1093/aje/kwr245 22025354

[6] Birrell PJ, Ketsetzis G, Gay NJ, Cooper BS, Presanis AM, Harris RJ, et al Bayesian modeling to unmask and predict influenza A/H1N1pdm dynamics in London. Proc Natl Acad Sci USA. 2011 11;108(45):18238–18243. 10.1073/pnas.1103002108 22042838 PMC3215054

[7] Strengell M, Ikonen N, Ziegler T, Julkunen I. Minor changes in the hemagglutinin of Influenza A(H1N1)2009 virus alter its antigenic properties. PLoS ONE. 2011 10;6(10):e25848 Available from: http://dx.doi.org/10.1371%2Fjournal.pone.0025848 10.1371/journal.pone.0025848 22022458 PMC3191144

[8] Dureau J, Kalogeropoulos K, Baguelin M. Capturing the time-varying drivers of an epidemic using stochastic dynamical systems. Biostatistics. 2013 7;14(3):541–555. 10.1093/biostatistics/kxs052 23292757

[9] Jacks A, Ollgren J, Ziegler T, Lyytikainen O. Influenza-associated hospitalisations in Finland from 1996 to 2010: unexpected age-specific burden during the influenza A(H1N1)pdm09 pandemic from 2009 to 2010. Euro Surveill. 2012;17(38). 23040966

[10] Mossong J, Hens N, Jit M, Beutels P, Auranen K, Mikolajczyk R, et al Social contacts and mixing patterns relevant to the spread of infectious diseases. PLoS Med. 2008 3;5(3):e74 10.1371/journal.pmed.0050074 18366252 PMC2270306

[11] Syrjanen RK, Jokinen J, Ziegler T, Sundman J, Lahdenkari M, Julkunen I, et al Effectiveness of pandemic and seasonal influenza vaccines in preventing laboratory-confirmed influenza in adults: a clinical cohort study during epidemic seasons 2009-2010 and 2010-2011 in Finland. PLoS ONE. 2014;9(9):e108538 10.1371/journal.pone.0108538 25265186 PMC4180439

[12] Diekmann O, Heesterbeek JAP. Mathematical epidemiology of infectious diseases: model building, analysis, and interpretation Wiley series in mathematical and computational biology. Chichester, New York: John Wiley; 2000 Available from: http://opac.inria.fr/record=b1096826

[13] Presanis aa A M. The severity of pandemic H1N1 influenza in the United States, from April to July 2009: a Bayesian analysis. PLoS Med. 2009 12;6:e1000207 10.1371/journal.pmed.1000207 19997612 PMC2784967

[14] Miller E, Hoschler K, Hardelid P, Stanford E, Andrews N, Zambon M. Incidence of 2009 pandemic influenza A H1N1 infection in England: a cross-sectional serological study. Lancet. 2010 3;375:1100–1108. 10.1016/S0140-6736(09)62126-7 20096450

[15] Reed C, Angulo FJ, Swerdlow DL, Lipsitch M, Meltzer MI, Jernigan D, et al Estimates of the prevalence of pandemic (H1N1) 2009, United States, April-July 2009. Emerging Infect Dis. 2009 12;15:2004–2007. 10.3201/eid1512.091413 19961687 PMC3375879

[16] Baker MG, Wilson N, Huang QS, Paine S, Lopez L, Bandaranayake D, et al Pandemic influenza A(H1N1)v in New Zealand: the experience from April to August 2009. Euro Surveill. 2009;14. 10.2807/ese.14.34.19319-en 19712648

[17] Andrieu C, Doucet A, Holenstein R. Particle Markov chain Monte Carlo methods. Journal of the Royal Statistical Society: Series B. 2010;p. 269–342. 10.1111/j.1467-9868.2009.00736.x

[18] Andrieu C, Roberts GO. The Pseudo-Marginal Approach for Efficient Monte Carlo Computations. The Annals of Statistics. 2009;37(2):pp. 697–725. Available from: http://www.jstor.org/stable/30243645 10.1214/07-AOS574

[19] Dorigatti I, Cauchemez S, Ferguson NM. Increased transmissibility explains the third wave of infection by the 2009 H1N1 pandemic virus in England. Proc Natl Acad Sci USA. 2013 8;110(33):13422–13427. 10.1073/pnas.1303117110 23882078 PMC3746905

[20] Earn DJD, Dushoff J, Levin SA. Ecology and evolution of the flu. Trends in Ecology & Evolution. 2002;17(7):334–340. Available from: http://www.sciencedirect.com/science/article/pii/S0169534702025028 10.1016/S0169-5347(02)02502-8

[21] Shaman J, Kohn M. Absolute humidity modulates influenza survival, transmission, and seasonality. Proceedings of the National Academy of Sciences. 2009;106(9):3243–3248. 10.1073/pnas.0806852106 PMC2651255 19204283

