# Supplementary material for: Revealing the True Incidence of Pandemic A(H1N1)pdm09 Influenza in Finland during the First Two Seasons — An Analysis Based on a Dynamic Transmission Model
Source: PLoS Comput Biol. 2016 Mar 24;12(3):e1004803. doi: 10.1371/journal.pcbi.1004803 (PMC4807082; doi:10.1371/journal.pcbi.1004803)
Supplement: S1 Appendix — (PDF) [file pcbi.1004803.s001.pdf]

Revealing the true incidence of pandemic A(H1N1)pdm09 influenza in Finland during the first two seasons

Mikhail Shubin, Artem Lebedev, Outi Lyytikäinen, Kari Auranen

Supplement 1: Contact rates

The contact rates in the population were estimated from the Finnish arm of the Polymod survey (Mossong et al., 2008). The data contain information about the daily contacts in a random sample of 1006 individuals in Finland. For each individual, information about the number and age of his/her contacts were used in this study. All contacts (with and without physical touch) were taken into account. The contact frequency was not taken into account (e.g. if a participant reported having 5 contacts with the same individual, we count a single contact).

Let R be the set of all observations such that R_{bi} is the total number of contacts reported by participant i to individuals from age group b, A_i the index of age group of individual i, $P_a = \sum_{i=1}^{1006} \mathbb{1}(A_i = a)$ the number of study participants in age group a, and $T_{ba} = \sum_{i=1}^{1006} R_{bi} \mathbb{1}(A_i = a)$ the total number of reported contacts from age group a to b.

The simplest way to construct a contact matrix would be to assign

$$C_{ba}^{\text{naive}} = \frac{T_{ba}}{P_a}.$$

However, C^{naive} is unsuitable because a) some of the elements of the matrix will be equal to zero, which is unrealistic, and b) the symmetry condition $N_a C_{ba} = N_b C_{ab}$ would not hold (here N is the population size).

To solve this problem, we estimate the contact rates as follows. Assume C_{ba} is the true contact matrix, agreeing with the constraint $N_a C_{ba} = N_b C_{ab}$. We chose the elements C_{ba} for $b \leq a$ to be the free parameters, and define the prior for them as

$$C_{ba} \sim \text{Exponential}\left(1 + N_a/N_b\right).$$

The prior is consistent under the transformation $C_{ab} = N_a/N_b C_{ba}$, i.e. the induced prior distribution would follow the same expression for C_{ba}, $b > a$.

We assume that the reported number of contacts is a random variable following a Poisson distribution:

$$R_{bi} \sim \text{Poisson}\left(C_{bA_i}\right) \text{ for all participants } i.$$

The likelihood of C_{ba}, $b < a$, then takes the following form:

$$\begin{aligned}
p(R|C_{ba}) &= \prod_{i,A_i=a} \text{Poisson}\left(R_{bi}|C_{ba}\right) \prod_{i,A_i=b} \text{Poisson}\left(R_{ai}|C_{ab}\right) \\
&= \prod_{i,A_i=a} \text{Poisson}\left(R_{bi}|C_{ba}\right) \prod_{i,A_i=b} \text{Poisson}\left(R_{ai}\middle|\frac{N_a}{N_b}C_{ba}\right) \\
&\propto C_{ba}^{T_{ba}} \exp(-C_{ba}P_a) \left(\frac{N_a}{N_b}C_{ba}\right)^{T_{ab}} \exp\left(-\frac{N_a}{N_b}C_{ba}P_b\right) \\
&\propto (C_{ba})^{T_{ba}+T_{ab}} \exp\left(-C_{ba}\left(P_a + \frac{N_a}{N_b}P_b\right)\right).
\end{aligned}$$

The posterior for C_{ba}, $a < b$, is then a Gamma distribution with the shape α and rate β:

$$p(C_{ba}|R) \sim \text{Gamma}\left(\alpha = T_{ba} + T_{ab} + 1, \beta = (P_a + 1) + \frac{N_a}{N_b}(P_b + 1)\right),$$

Similarly, for the case $a = b$ the posterior distribution takes following form:

$$p(C_{aa}|R) \sim \text{Gamma}\left(\alpha = T_{aa} + 1, \beta = P_a + 2\right).$$

In the transmission model, we use the posterior mean (evaluated as α/β) multiplied by 7 to scale daily contacts into weekly were used.

$$\begin{aligned}
C_{a\to b} &= 7\frac{T_{ba} + T_{ab} + 1}{(P_a + 1) + \frac{N_a}{N_b}(P_b + 1)}, a < b \\
C_{a\to a} &= 7\frac{T_{aa} + 1}{P_a + 2}
\end{aligned}$$

The Polymod survey contain several non-complete records. The missing data was handled in the following way: 2 out of 1006 participants have their age missing and were ignored; If age of the contactee is given as an interval (e.g. between 30 and 40 year old), the mean was used to determine his/her age group; if the age of the contactee is given as the upper bound, the upper bound was used (e.g. if data tells that contactee is less then 20 years old, we took it as 20).

References

Mossong, J., Hens, N., Jit, M., Beutels, P., Auranen, K., Mikolajczyk, R., Massari, M., Salmaso, S., Tomba, G. S., Wallinga, J., Heijne, J., Sadkowska-Todys, M., Rosinska, M., and Edmunds, W. J. (2008). Social contacts and mixing patterns relevant to the spread of infectious diseases. *PLoS Med.*, 5(3):e74.
