# Supplementary material for: Revealing the True Incidence of Pandemic A(H1N1)pdm09 Influenza in Finland during the First Two Seasons — An Analysis Based on a Dynamic Transmission Model
Source: PLoS Comput Biol. 2016 Mar 24;12(3):e1004803. doi: 10.1371/journal.pcbi.1004803 (PMC4807082; doi:10.1371/journal.pcbi.1004803)
Supplement: S2 Appendix — (PDF) [file pcbi.1004803.s002.pdf]

Revealing the true incidence of pandemic A(H1N1)pdm09 influenza in Finland during the first two seasons

Mikhail Shubin, Artem Lebedev, Outi Lyytikäinen, Kari Auranen

Supplement 2: The model of influenza transmission and disease

The model is defined by the following set of equations:

$$
\begin{aligned}
S_{a,0} &= N_a \\
I_{a,0} &= 0 \\
r_{a,t} &= 1 - (1 - q_a)\left(1 - \frac{p_a}{N_a}\right)^{w_t \sum_{b=1}^{16} C_{b \to a} I_{b,t-1}} \\
I_{a,t} &\sim \text{Binom}\left(S_{a,t-1}; r_{a,t}\right) \\
S_{a,t} &\sim \text{Binom}\left(S_{a,t-1} - I_{a,t}; 1 - v_{a,t}\right) \\
I_{a,t}^{(\text{severe})} &\sim \text{Binom}(I_{a,t}; s_a^{(\text{sev/inf})}) \\
I_{a,t}^{(\text{IC})} &\sim \text{Binom}(I_{a,t}^{(\text{severe})}; s_a^{(\text{IC/sev})}) \\
I_{a,t}^{(\text{mild})} &= I_{a,t} - I_{a,t}^{(\text{severe})} \\
I_{a,t}^{(\text{hosp})} &= I_{a,t}^{(\text{severe})} - I_{a,t}^{(\text{IC})} \\
D_{a,t}^{(\text{mild})} &\sim \text{Binom}\left(I_{a,t}^{(\text{mild})}; d_t^{(\text{mild})}\right) \\
D_{a,t}^{(\text{hosp})} &\sim \text{Binom}\left(I_{a,t}^{(\text{hosp})}; d^{(\text{hosp})} \mathbb{1}(t < 60 \text{ weeks})\right) \\
D_{a,t}^{(\text{IC})} &= I_{a,t}^{(\text{IC})} \\
w_t &= 2 \text{ logistic}(\varepsilon_t) \\
d_t^{(\text{mild})} &= \text{ logistic}(\sigma_t/\sqrt{0.01} + \text{ logit}(0.01))
\end{aligned}
$$

(The equations for $I_{a,t}$ and $S_{a,t}$ are bracketed as 1; the equations for $I_{a,t}^{(\text{severe})}$, $I_{a,t}^{(\text{IC})}$, $I_{a,t}^{(\text{mild})}$ and $I_{a,t}^{(\text{hosp})}$ are bracketed as 2.)

For notation, see the main text. Figure 1 presents the model as a directed acyclic graph.

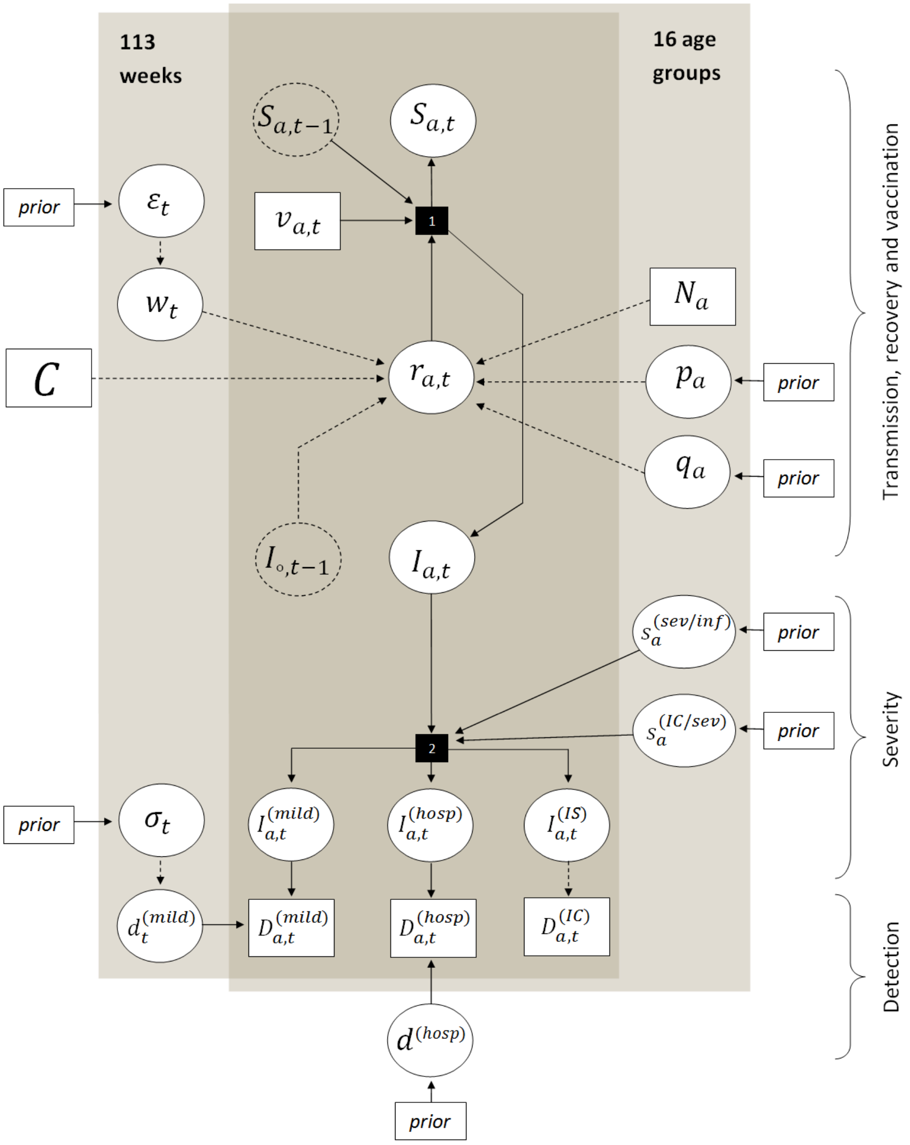

Figure 1: **DAG of the model.** Circles represent model unknowns, rectangles known or fixed values. The plates highlight the values specified for each week and/or age group. Dotted circles are used to show the relations between strata. Smaller rectangles with "prior" sign point out those model parameters with specified prior distributions. Stochastic relations are indicated with solid lines, deterministic with dashed lines. Complex relations are shown as black rectangles and and written out in the model equations.

Weekly time steps. We used a discrete-time dynamical model with the time step taken to be one week. A model with a longer time step would miss some features of the weekly aggregated data. A model with a shorter time step could be more accurate but also computationally more expensive due to a large number of hidden model states. In Supplement S6 Appendix we show that the discrete-time SIR model with the same next generation function agrees well with the continuous-time SIR with the same R_0.

Stratification. We stratified the data into 16 age groups. To improve the identifiability of the model parameters, we grouped parameters p, q, $s^{(\text{sev/inf})}$ and $s^{(\text{IC/sev})}$ further into 6 bigger strata: 0-4, 5-14, 15-19, 20-29, 30-64, 65+ years of age.

We assumed the detection probabilities $d_t^{(\text{mild})}$ and $d^{(\text{hosp})}$ to be independent of age. This choice was based on the previous study, where we had not found evidence for strong variation across age groups (range 3.1 – 4.6%). By contrast, the previous analysis discovered significant variation across regions (range 1.3 – 6.9%), but the current model excluded any between-region variance altogether for computational reasons.

Prior formulation. In the previous study, we used conjugate beta-distributed priors (Shubin et al., 2014). For this model we used log-normal and logit-normal priors for the same parameters to represent the same information (see Table 1 in the main text). These prior are less heavy tailed, i.e. more informative.

Derivation of the infection pressure. Assume that a population of size N receives a single contact from an infectious host. The probability for a susceptible individual in this population to receive this contact is $1/N$. The probability of escaping infection at this contact is $1 - p/N$. If the population receives M independent contacts from infectious hosts, then the probability for one susceptible individual to escape infection is $(1 - p/N)^M$. In addition, there is a probability q to acquire infection from outside the population. The probability for a single individual to become infected is thus $1 - (1 - q)(1 - p/N)^M$.

For age group a at week t, the total number of contacts from all infectious hosts in the population is calculated as $M = \sum_{b=1}^{16} C_{b\to a} I_{b,t-1}$. This number is then scaled by the transmission random effect w_t, leading to the expression of $r_{a,t}$ in the model equations.

Derivation of basic reproduction number The basic reproduction number R_0 (the number of secondary infection caused by a single infected individuals in a totally susceptible population) can be evaluated as a largest eigenvalue of a next generation matrix NGM (Diekmann and Heesterbeek,

2000). The element of the next generation matrix NGM_{ab} is defined as a mean number infections caused in the age group a by an infected individual from the age group b. In our model this number is equal to

$$S_{a,t}\left(1-\left(1-\frac{p_a}{N_a}\right)^{w_t C_{b\to a}}\right).$$

As we assume that the population is completely susceptible ($S_{a,t} = N_a$) and the transmission random effect is at its mean value ($w_t = 1$), the mean number of new infections is

$$NGM_{ba} = N_a\left(1-\left(1-\frac{p_a}{N_a}\right)^{C_{b\to a}}\right).$$

While $p_a/N_a \to 0$, this expression can be approximated with

$$NGM_{ba} \approx N_a\left(1-\left(1-\frac{p_a}{N_a}C_{b\to a}\right)\right) = p_a C_{b\to a}.$$

The number of secondary infections caused by an infectious individual from age group b in the whole population is then approximated with $\sum_{a=1}^{16} NGM_{ab} = \sum_{a=1}^{16} p_a C_{b\to a}$.

At each week, there is chance of being infected from outside the population. The mean number of such infections in a totally susceptible population is $N_a q_a$.

References

Diekmann, O. and Heesterbeek, J. A. P. (2000). *Mathematical epidemiology of infectious diseases : model building, analysis, and interpretation.* Wiley series in mathematical and computational biology. John Wiley, Chichester, New York.

Shubin, M., Virtanen, M., Toikkanen, S., Lyytikainen, O., and Auranen, K. (2014). Estimating the burden of A(H1N1)pdm09 influenza in Finland during two seasons. *Epidemiol. Infect.*, 142(5):964–974.
