# Supplementary material for: Revealing the True Incidence of Pandemic A(H1N1)pdm09 Influenza in Finland during the First Two Seasons — An Analysis Based on a Dynamic Transmission Model
Source: PLoS Comput Biol. 2016 Mar 24;12(3):e1004803. doi: 10.1371/journal.pcbi.1004803 (PMC4807082; doi:10.1371/journal.pcbi.1004803)
Supplement: S3 Appendix — (PDF) [file pcbi.1004803.s003.pdf]

Revealing the true incidence of pandemic A(H1N1)pdm09 influenza in Finland during the first two seasons

Mikhail Shubin, Artem Lebedev, Outi Lyytikäinen, Kari Auranen

Supplement 3: Computational methods

The computational problem is to sample from the posterior distribution $p(\theta, H|D)$. Here θ denotes the unknown model parameters:

$$\theta = \{p, q, s^{(\text{sev/inf})}, s^{(\text{IC/sev})}, d^{(\text{hosp})}, \varepsilon, \sigma\}$$

H denotes the hidden model states (the age group specific weekly numbers of infectious and susceptible individuals):

$$H = \{I_{a,t}, S_{a,t}\}_{a \in 1\ldots16;\ t \in 0\ldots112}$$

and D denotes the observations (i.e. the age group specific weekly numbers of detected cases):

$$D = \{D^{(\text{mild})}_{a,t}, D^{(\text{hosp})}_{a,t}, D^{(\text{IC})}_{a,t}\}_{a \in 1\ldots16;\ t \in 0\ldots112}.$$

Above a is the age group index and t is the week index. We solve the computational problem using the following algorithms:

- Markov chain Monte Carlo (MCMC). It was used as a main inference tool.
- Iterative component-wise optimization. Auxiliary methods. Used to find the maximum of the posterior. The maximum is then used as the initial point for MCMC. Optimization was also used for the sensitivity analysis.
- Exact approximate MCMC (Andrieu and Roberts, 2009) targeting the tempered marginal posterior distributions $p(\theta|D)^{\frac{1}{5}}$ and $p(\theta|D)^{\frac{1}{25}}$. Auxiliary methods. The tempered posteriors were studied to ensure that the peak area of the target posterior is unimodal and well-behaving.

- Importance sampler targeting the likelihood $p(D|\theta)$. Auxiliary methods, used within Exact approximate MCMC.

The following text gives an overview of the computational methods. All probabilities and importance weights were log-transformed. The model parameters p, $s^{(\text{sev/inf})}$, $s^{(\text{IC/sev})}$, $d^{(\text{hosp})}$ were explored in a logit form. Parameter q was explored in a log form. The model parameters w_t and $d_t^{(\text{mild})}$ were deterministicly transformed parameters ε and σ, and therefore not taken into analysis explicitly.

We use the following notation: $\mathcal{N}(\mu, \sigma^2)$ is a normal distribution with mean μ and variance σ^2; $\mathbb{N}(\overline{\mu}, \Sigma)$ is a multivariate normal distribution with mean $\overline{\mu}$ and covariance matrix Σ; $U(0,1)$ is a number sampled uniformly from the interval $(0,1)$, '$\leftarrow$' is an assignment operator.

MCMC

We used MCMC as a main inference tool targeting the full posterior of the model unknowns $p(\theta, H|D)$. The chain was initiated with the maximum estimated with optimization (see below). The parameters ε, σ and the hidden states H were updated in two different ways during the odd and even iterations to improve mixing.

The model parameters p, q, $s^{(\text{sev/inf})}$, $s^{(\text{IC/sev})}$ were updated using the following Metropolis-Hastings steps:

1. The proposed value for p is sampled in logit form

$$\text{logit}(p^*) \sim \mathbb{N}(\text{logit}(p), \Sigma_p),$$

here Σ_p is the proposal covariance matrix for the parameter logit(p).

2. The proposal is accepted $(p \leftarrow p^*)$ if

$$U(0,1) < \frac{p(D, H|p^*, \dots)\pi(\text{logit}(p^*))}{p(D, H|p, \dots)\ \pi(\text{logit}(p))};$$

here $\pi()$ is a prior for logit(p).

3. The parameters q, $s^{(\text{sev/inf})}$ and $s^{(\text{IC/sev})}$ were updated in the same way using steps 1-2. For the parameter q the log transformation is used instead of logit.

The detection probability for a hospitalized case $d^{(\text{hosp})}$ was updated using the following Metropolis-Hastings steps:

1. The proposed values are sampled in logit form

$$\text{logit}(d^{(\text{hosp})*}) \sim \mathcal{N}(\text{logit}(d^{(\text{hosp})}), \sigma^2_{d^{(\text{hosp})}})$$

here $\sigma^2_{d^{(\text{hosp})}}$ is the proposal variance.

2. The proposal is accepted ($d^{(\text{hosp})} \leftarrow d^{(\text{hosp})*}$) if

$$U(0,1) < \frac{p\left(D, H | d^{(\text{hosp})*} \dots\right)}{p\left(D, H | d^{(\text{hosp})} \dots\right)} \times \frac{\pi\left(\text{logit}(d^{(\text{hosp})*})\right)}{\pi\left(\text{logit}(d^{(\text{hosp})})\right)}$$

here $\pi()$ is a prior for $\text{logit}(d^{(\text{hosp})})$.

On the odd iterations, parameters ε, σ were updated using the following Metropolis-Hastings steps:

1. Randomly sample two integers i and j, such that $0 < i < j < T$, here $T = 113$ weeks is length of the studied period.

2. Sample a proposal for the middle part of the vector $\varepsilon_{i \dots j}$ from the conditioned prior (i.e. a conditioned multivariate normal distribution):
$$\varepsilon^*_{i \dots j} \sim \pi(\varepsilon_{i \dots j} | \varepsilon_{0 \dots i-1, j+1 \dots T})$$

3. The proposal is accepted ($\varepsilon_{i \dots j} \leftarrow \varepsilon^*_{i \dots j}$) if

$$U(0,1) < \frac{p(D, H | \varepsilon_{0 \dots i-1}, \varepsilon^*_{i \dots j}, \varepsilon_{j+1 \dots T-1}, \dots)}{p(D, H | \varepsilon_{0 \dots T-1}, \dots)}.$$

4. The head and tail parts of the vector ε in the same way using the step 3:

$$\varepsilon^*_{0 \dots i-1} \sim \pi(\varepsilon_{0 \dots i-1} | \varepsilon_{i \dots T-1});$$
$$\varepsilon^*_{j+1 \dots T-1} \sim \pi(\varepsilon_{j+1 \dots T-1} | \varepsilon_{0 \dots j}).$$

5. The parameter σ is updated in the same way using steps 1-3.

On the even iterations, parameters ε, σ were updated using the following

approximation to the Metropolis-Hastings steps.

1. The proposed value is sampled

$$\varepsilon^* \sim \mathbb{N}(\varepsilon, \Sigma_\varepsilon),$$

here Σ_ε is a proposal covariance matrix for the parameter ε.

2. The first component of the proposal is accepted ($\varepsilon_0 \leftarrow \varepsilon_0^*$) if

$$U(0,1) < \frac{p(D,H|\varepsilon_0^*, \varepsilon_{1\ldots T}, \ldots)\pi(\varepsilon_0^*, \varepsilon_{1\ldots T})}{p(D,H|\varepsilon_{0\ldots T}, \ldots)\pi(\varepsilon_{0\ldots T})},$$

here $\pi()$ is the prior.

3. The second component of the proposal is accepted ($\varepsilon_1 \leftarrow \varepsilon_1^*$) if

$$U(0,1) < \frac{p(D,H|\varepsilon_0, \varepsilon_1^*, \varepsilon_{2\ldots T}, \ldots)\pi(\varepsilon_0, \varepsilon_1^*, \varepsilon_{2\ldots T})}{p(D,H|\varepsilon_{0\ldots T}, \ldots)\pi(\varepsilon_{0\ldots T})}.$$

4. The process is sequentially repeated for all components of ε.

5. The parameter σ us updated in the same way using steps 1-4.

On the odd iterations, hidden states were updated using a particle Gibbs sampler (Andrieu et al., 2010) step with $n = 200$ particles. The importance function used in the Gibbs step is described in the Importance Sampler section.

1. Initialize the set of n particles

$$\left\{\{I_{a,0}^{(i)} = 0, S_{a,0}^{(i)} = N_a\}_{a\in 1\ldots 16}\right\}_{i\in 1\ldots n}$$

Initialize importance weights:

$$\left\{W_{a,0}^{(i)} = 1\right\}_{i\in 1\ldots n}$$

2. Propagate the particles

$$H_t^{(i)} \sim g(H_{t-1}^{(i)}, D, \theta) \text{ for } i \in 2\ldots n$$

here $H_t^{(i)} = \left\{I_{a,t}^{(i)}, S_{a,t}^{(i)}\right\}_{a \in 1 \ldots 16}$ and g is the importance function. The first particle plays the role of a reference. It is not sampled randomly, but is set to be equal to the hidden state H from the current state of the chain:

$$H_t^{(1)} = H_t$$

3. Propagate the importance weights

$$W_t^{(i)} = W_{t-1}^{(i)} \frac{p(D, H_t^{(i)} | H_{t-1}^{(i)}, \theta)}{g(H_t^{(i)} | H_{t-1}^{(i)}, D, \theta)} \text{ for } i \in 1 \ldots n$$

4. During iterations $t \in 20, 25, 30 \ldots 110$, after the particle and weights are propagated, conduct resampling. Resample the set of particles using their normalized weights. Keep the first (reference) particle intact.

$$ind_i = \text{Categorical}\left(\frac{W_t^{(1)}}{\sum_j W_t^{(j)}}, \frac{W_t^{(2)}}{\sum_j W_t^{(j)}} \cdots \frac{W_t^{(n)}}{\sum_j W_t^{(j)}}\right)$$
$$H_{0 \ldots t}^{(i)} \leftarrow H_{0 \ldots t}^{(ind_i)} \quad \text{for } i \in 2 \ldots n$$
$$W_t^{(i)} \leftarrow 0 \quad \text{for } i \in 1 \ldots n$$

Resampling is done using a resampling scheme (Douc et al., 2005)

5. After all weeks are sampled, select one of the particles using the normalized weights to be the proposal. Proposal is always accepted.

$$proposal = \text{Categorical}\left(\frac{W_T^{(1)}}{\sum_j W_T^{(j)}}, \frac{W_T^{(2)}}{\sum_j W_T^{(j)}} \cdots \frac{W_T^{(n)}}{\sum_j W_T^{(j)}}\right)$$
$$H_{0 \ldots T} \leftarrow H_{0 \ldots T}^{(proposal)}$$

On the even iterations, hidden states were updated using the algorithm:

1. Sample a proposal for the hidden states of the first age strata,

keeping the rest of the hidden states intact:

$$I_1^*, S_1^* \sim g(I_{2\dots16}, S_{2\dots16}, D, \theta);$$

Here $I_a = \{I_{a,t}\}_{t\in 0\dots T}$, $S_a = \{S_{a,t}\}_{t\in 0\dots T}$ and g is the importance function.

2. The proposal is accepted ($I_1 \leftarrow I_1^*$, $S_1 \leftarrow S_1^*$) if

$$U(0,1) < \frac{p(D, I_1^*, S_1^*, I_{2\dots16}, S_{2\dots16}|\theta)}{p(D, I_{1\dots16}, S_{1\dots16}|\theta)} \frac{g(I_1, S_1|I_{2\dots1,}, S_{2\dots16}, D, \theta)}{g(I_1^*, S_1^*|I_{2\dots16}, S_{2\dots16}, D, \theta)}.$$

3. Each other stratum is updated in the same way using steps 1-2 .

We used 15 independently run chains, each with 55000 iteration, discarding the initial 15000 iterations as warm-up and recording every 20th iteration. Each iteration included updating p, q, $s^{(\text{sev/inf})}$, $s^{(\text{IC/sev})}$, $d^{(\text{hosp})}$, ε, σ and the hidden states. Proposal variances Σ and σ^2 were adopted during the warm-up phase to obtain an approximate acceptance rate of 1/3 (Brooks et al., 2011).

Optimization

Before starting the MCMC simulation to explore the posterior, the maximum of the posterior first was searched by an *ad hoc* optimization algorithm. The MCMC chains were then initiated with the found maximum point. In the sensitivity analyses, the results were compared in terms of the posterior maximum points found by the algorithm. The optimization is initiated at a random point. For each parameter $x \in \theta$ or hidden value $x \in H$ the proposal distance $step_x$ is set to the arbitrary non-zero value. Parameters and hidden values are updated in a slightly different way because all parameters are continuous while all hidden values are discrete.

The hidden values $x \in H$ are updated in the following way:

1. Let x_0 denote the current value of the hidden value x. If $x = 0$, set $step_x = 1$

2. A new value for x is proposed:

$$x^* = max(0, x_0 + step_x)$$

3. The part of the posterior density that depends on x is evaluated for the two values of x:

$$P_0 \sim p(x_0, \dots |D)$$
$$P^* \sim p(x^*, \dots |D)$$

4. if $P^0 > P^*$, keep the value $x = x_0$ and set

$$step_x \leftarrow round(-0.5\ step_x + 0.6)$$

5. if $P^* > P_0$, set the value $x \leftarrow x^*$ and set

$$step_x \leftarrow round(+1.6\ step_x)$$

The parameters $x \in \theta$ are updated in the following way:

1. Let x_0 denotes the current value of the hidden value x. Two new values for x are proposed:

$$x^* = x_0 + step_x$$
$$x^{**} = x_0 + 2\ step_x$$

2. The part of the posterior depending on x is evaluated for the different values of x:

$$P_0 \sim p(x_0, \dots |D)$$
$$P^* \sim p(x^*, \dots |D)$$
$$P^{**} \sim p(x^{**}, \dots |D)$$

3. if $P^0 > P^*, P^{**}$ keep the value $x = x_0$ and set

$$step_x \leftarrow -0.5\ step_x$$

4. if $P^* > P_0, P^{**}$ set the value $x \leftarrow x^*$;

   (a) if $P_0 = P^{**}$, set $step_x \leftarrow +0.1\ step_x$;

   (b) else, set $step_x \leftarrow \dfrac{P^{**} - P_0}{2\ max(P^* - P^{**}, P^* - P_0)} step_x$

5. if $P^{**} > P_0, P^*$ set the value $x \leftarrow x^{**}$; set

$$step_x \leftarrow \frac{P^{**} - P_0}{max(0.1\ (P^{**} - P_0), P^* - P_0)} step_x$$

6. if $|step_x| < 0.001$, set $step_x \leftarrow 0.001\ sign(step_x)$

We run the optimization algorithm for 5000 iterations.

Exact approximate MCMC

We used Exact approximate MCMC (Andrieu and Roberts, 2009) to target the tempered marginal posterior $p(\theta|D)^{1/25}$. The chain was initiated with the maximum estimated with the optimization (see above). The algorithm proceeded by sequentially updating the components of θ. The sampler was used twice with the temperature set to $temp = 1/25$ and $temp = 1/5$.

The model parameters p, q, $s^{(\mathrm{sev/inf})}$, $s^{(\mathrm{IC/sev})}$ were updated using the following Metropolis-Hastings steps:

1. The proposed value of p is sampled at the logit scale:

$$\mathrm{logit}(p^*) \sim \mathbb{N}(\mathrm{logit}(p), \Sigma_p),$$

here Σ_p is the proposal covariance matrix for $\mathrm{logit}(p)$.

2. The likelihood for the proposal is estimated with importance sampling

$$L^* \leftarrow \tilde{p}(D|p^*, \dots).$$

3. The proposal is accepted ($p \leftarrow p^*$; $L \leftarrow L^*$) if

$$U(0,1) < \left(\frac{L^* \pi(\mathrm{logit}(p^*))}{L\ \pi(\mathrm{logit}(p))} \right)^{temp},$$

here $\pi()$ is a prior for a logit form of p.

4. The parameters q, $s^{(\mathrm{sev/inf})}$ and $s^{(\mathrm{IC/sev})}$ are updated in the same way using steps 1-3. For the parameter q the log transformation is used instead of logit.

The detection probability for the hospitalized cases $d^{(\mathrm{hosp})}$ was updated using the following Metropolis-Hastings steps:

1. The proposed values are sampled in logit form
$$\text{logit}(d^{(\text{hosp})*}) \sim \mathcal{N}(\text{logit}(d^{(\text{hosp})}), \sigma^2_{d^{(\text{hosp})}}),$$
here $\sigma^2_{d^{(\text{hosp})}}$ is the proposal variance.
2. The likelihood for the proposal is estimated with importance sampling
$$L^* \leftarrow \tilde{p}(D|d^{(\text{hosp})*}, \dots).$$
3. The proposal is accepted ($d^{(\text{hosp})} \leftarrow d^{(\text{hosp})*}$; $L \leftarrow L^*$) if
$$U(0,1) < \left(\frac{L^* \pi\left(\text{logit}(d^{(\text{hosp})*})\right)}{L\ \pi\left(\text{logit}(d^{(\text{hosp})})\right)}\right)^{temp},$$
here $\pi()$ is a prior for a logit form of $d_t^{(\text{mild})}$.

The parameters ε, σ (determining random effects w and d) were updated using the algorithm:

1. Randomly sample two integers i and j, such that $0 < i < j < T$, here $T = 113$ here $T = 113$ weeks is length of the studied period.
2. Sample new proposal for the middle part of the vector $\varepsilon_{i\dots j}$ from the conditioned prior (i.e. conditioned multivariate normal distribution):
$$\varepsilon^*_{i\dots j} \sim \pi(\varepsilon_{0\dots i-1, j+1\dots T})$$
3. The likelihood for the proposal is estimated with importance sampling
$$L^* \leftarrow \tilde{p}(D|\varepsilon_{0\dots i-1}, \varepsilon^*_{i\dots j}, \varepsilon_{j+1\dots T}, \dots).$$
4. The proposal is accepted ($\varepsilon_{i\dots j} \leftarrow \varepsilon^*_{i\dots j}$; $L \leftarrow L^*$) if
$$U(0,1) < \left(\frac{L^*}{L}\right)^{temp}.$$
5. Steps 3-4 are repeated for the head and tail parts of the vector ε:
$$\varepsilon^*_{0\dots i-1} \sim \pi(\varepsilon_{i\dots T});$$
$$\varepsilon^*_{j+1\dots T} \sim \pi(\varepsilon_{0\dots j}).$$

6. The parameter σ is updated in the same way using steps 1-5.

We drew 6000 samples, discarding the initial 2000 as warm-up. Each iteration includes updating p, q, $s^{(\mathrm{sev/inf})}$, $s^{(\mathrm{IC/sev})}$, $d^{(\mathrm{hosp})}$, ε and σ. At the end of each non warm-up iteration the parameter vector was saved as one sample from the posterior $p(\theta|D)^{temp}$. The proposal variances Σ and σ^2 were adopted during the warm-up phase to get close the optimal acceptance rate 1/3 (Brooks et al., 2011).

Importance Sampler

We used an importance sampler to estimate the likelihood $p(D|\theta)$:

$$p(D|\theta) \approx \tilde{p}(\theta|D) = \frac{1}{n}\sum_{i=1}^{n}\frac{p(D, H_i|\theta)}{g(H_i|D,\theta)}; \qquad H_i \sim g(H_i|D,\theta).$$

Here the importance function $g()$ samples the hidden states exactly as defined by the model equations (see Supplement 2 Appendix) with an exception of the true numbers of infections $\{I_{a,t}\}_{a\in 1\ldots 16;\ t\in 0\ldots 112}$, which are sampled conditional on the data from the corresponding week:

$$\begin{aligned}
u_{a,t} &= r_{a,t}\left((1-s_a)(1-d_t^{(\mathrm{mild})}) + s(1-g_a)(1-d^{(\mathrm{hosp})})\right);\\
U_{a,t} &\sim \mathrm{Binom}\left(S_{a,t-1} - \left(D_{a,t}^{(\mathrm{mild})} + D_{a,t}^{(\mathrm{hosp})} + D_{a,t}^{(\mathrm{IC})}\right), \frac{u_{a,t}}{u_{a,t} + (1-r_{a,t})}\right),\\
I_{a,t} &\leftarrow U_{a,t} + D_{a,t}^{(\mathrm{mild})} + D_{a,t}^{(\mathrm{hosp})} + D_{a,t}^{(\mathrm{IC})}.
\end{aligned}$$

Here $U_{a,t}$ is the number of unobserved infections in age group a at week t, $r_{a,t}$ is the probability of becoming infected, evaluated according to the model equations; $u_{a,t}$ is the probability of becoming infected, but not detected.

Hardware and Software

The most computationally expensive procedure was the estimation of $p\,(D|\theta)$ with importance sampler. It was written in C and supports parallel computations on heterogeneous NUMA-systems comprising traditional multicore CPUs and Intel Xeon PHI accelerators. The idea to improve performance of the importance sampler was to distribute fixed number of Monte Carlo samples among computational threads assuming that CPUs composing NUMA were identical and accelerators were identical too. We run one worker for all host CPU cores and one worker for each Xeon PHI accelerator. Workers running on CPU cores and Xeon PHI cores have different efficiency,

so work distribution proportion for system with Q accelerators should be $W^{CPU} : W_1^{ACC} : ... : W_Q^{ACC} \approx \alpha : 1 : ... : 1$, where $W^{CPU} + QW_*^{ACC} = N$ assuming N is the total number of Monte Carlo casts.

We made estimations for $b = 10^5$ Monte Carlo casts and compute the proportion empirically: $\alpha = \frac{T_b^{ACC}}{T_b^{CPU}}$, where T_b^{CPU} – time to complete b Monte Carlo casts using CPU worker, T_b^{ACC} – time to complete b Monte Carlo casts using Xeon PHI worker, $W_*^{ACC} = \left\lfloor \frac{N}{\alpha+Q} \right\rfloor$ – number of Monte Carlo casts for each Xeon PHI worker, $W^{CPU} = N - Q \left\lfloor \frac{N}{\alpha+Q} \right\rfloor$ – number of Monte Carlo casts for CPU worker.

Every worker uses OpenMP to expose parallelism of the loop doing Monte Carlo casts. We used offload acceleration model to support Intel Xeon PHI capabilities. The code performing Monte Carlo cast is the same for both microarchitectures (x86 and MIC). Since we run bunch of separate computational threads, we need bunch of PRNG sequences considered to be uncorrelated to get necessary source of randomness (dedicated sequence for each thread). We use Intel MKL Mersenne Twister generator VSL_BRNG_MT2203 with 6024 pregenerated sequences designed specifically for massively parallel computations (this implies limit on parallelism – sampler can not utilize more than 6024 computational cores).

The rest of the code was written in Python 2.7 with external libraries `NumPy` and `SciPy` for mathemathical function and `Pillow` for visualization. All algorithms were executed on the RSATU server. The main MCMC algorithm took 1 month. The auxiliary MCMC took about 3 days each. The optimization required 2 days. RSATU server is NUMA-machine with two eight-core Intel Xeon E5-2690 CPUs (total 32 threads with Hyper-Threading) and one Intel Xeon PHI 3100 accelerator with 57 cores (total 228 threads – each core supports four hardware threads). The machine had 64Gb DDR3 RAM and the accelerator had 6Gb of on-board GDDR5 RAM. We run 32+228=250 threads to make computations and utilize 250 PRNG sequences accordingly. The proportion of work distribution was $\approx 1.6 : 1$.

References

Andrieu, C., Doucet, A., and Holenstein, R. (2010). Particle markov chain monte carlo methods. *Journal of the Royal Statistical Society: Series B*, pages 269–342.

Andrieu, C. and Roberts, G. O. (2009). The pseudo-marginal approach for efficient monte carlo computations. *The Annals of Statistics*, 37(2):pp. 697–725.

Brooks, S., Gelman, A., Jones, G. L., and Meng, X.-L. (2011). *Handbook of Markov Chain Monte Carlo*. Chapman and Hall/CRC.

Douc, R., Cappé, O., and Moulines, E. (2005). Comparison of resampling schemes for particle filtering. *CoRR*, abs/cs/0507025.
