# Supplementary material for: Revealing the True Incidence of Pandemic A(H1N1)pdm09 Influenza in Finland during the First Two Seasons — An Analysis Based on a Dynamic Transmission Model
Source: PLoS Comput Biol. 2016 Mar 24;12(3):e1004803. doi: 10.1371/journal.pcbi.1004803 (PMC4807082; doi:10.1371/journal.pcbi.1004803)
Supplement: S4 Appendix — (PDF) [file pcbi.1004803.s004.pdf]

Revealing the true incidence of pandemic A(H1N1)pdm09 influenza in Finland during the first two seasons

Mikhail Shubin, Artem Lebedev, Outi Lyytikäinen, Kari Auranen

Supplement 4: Additional results

The pictures in this Supplement illustrate additional results: posterior distribution of the parameters $d^{(\text{hosp})}$, $d_t^{(\text{mild})}$, w_t; realised reproduction number R_t; detection ratio per week; posterior distribution of the number of infections by age group; correlation between the parameters. All distributions are visualized with more probable values represented by more concentrated color. In addition, a few samples from the distributions are shown.

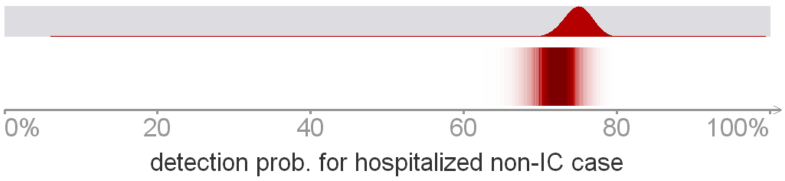

Figure 1: **The posterior distribution of the detection probability for the hospitalized cases $d^{(\text{hosp})}$.** The prior distribution for the $d^{(\text{hosp})}$ is shown for reference.

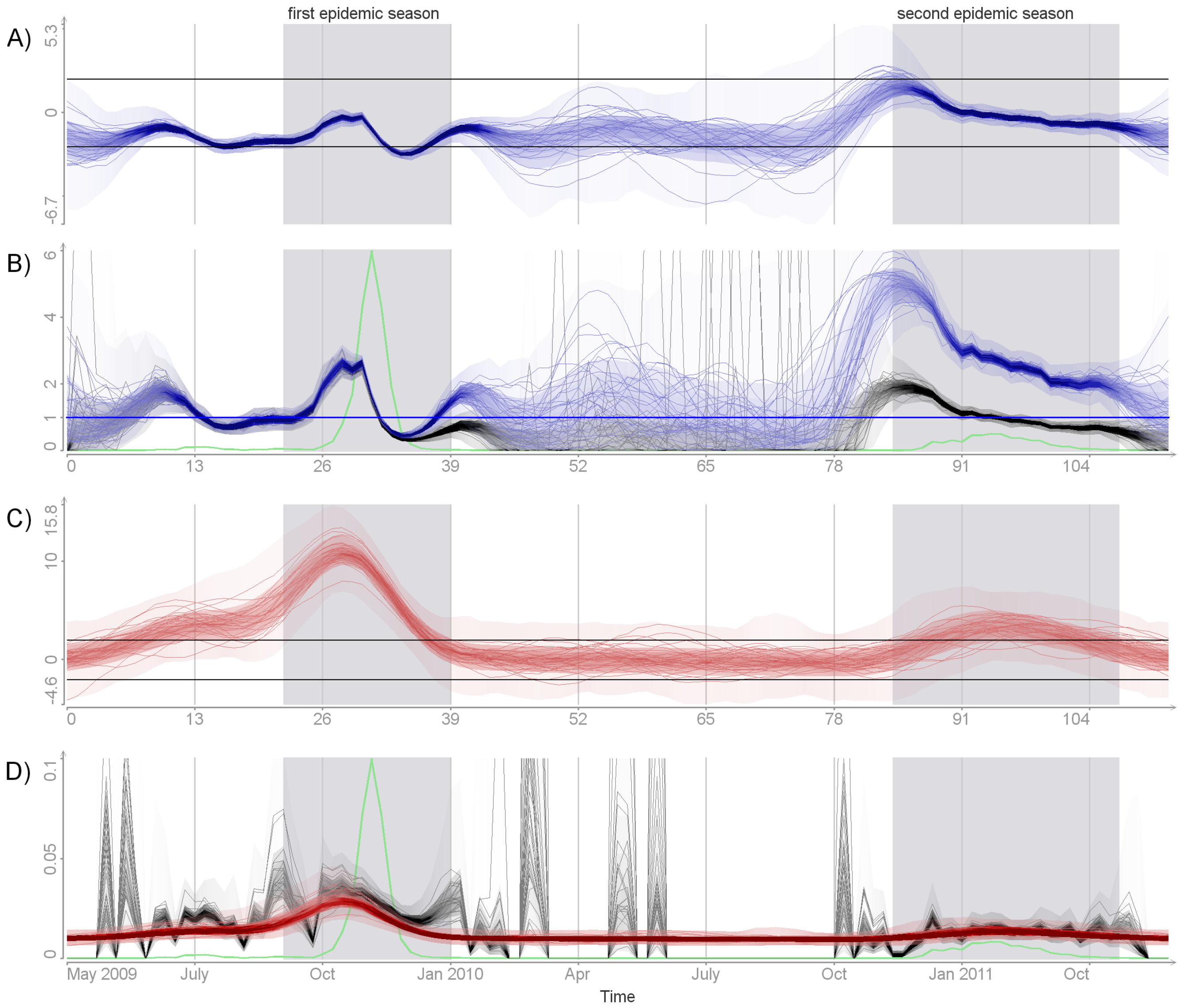

Figure 2: **The posterior distribution of the time-dependent variables.** Panel A: parameter ε_t (transformed w_t). The black horizontal lines mark 95% prior credible interval. Panel B: Basic reproduction number $R_{0,t}$ (blue lines, duplicated from the main text) and the realized reproduction number R_t (black lines). The realized R_t was estimated as $I_t/(I_{t-1}-\sum q_a S_a)$ for $(I_{t-1}-\sum q_a S_a) > 0$. The denominator represent the number of new infections introduced through within-population transmission. The green lines show the numbers of observed cases (not to scale). Panel C: parameter δ_t (transformed $d_t^{(\mathrm{mild})}$). The black horizontal lines mark 95% prior credible interval. Panel D: detection probability for the mild cases $d_t^{(\mathrm{mild})}$ (red lines, duplicated from the main text) and the detection ratio D_t/I_t (black lines). Green line shows the numbers of observed cases (not to scale).

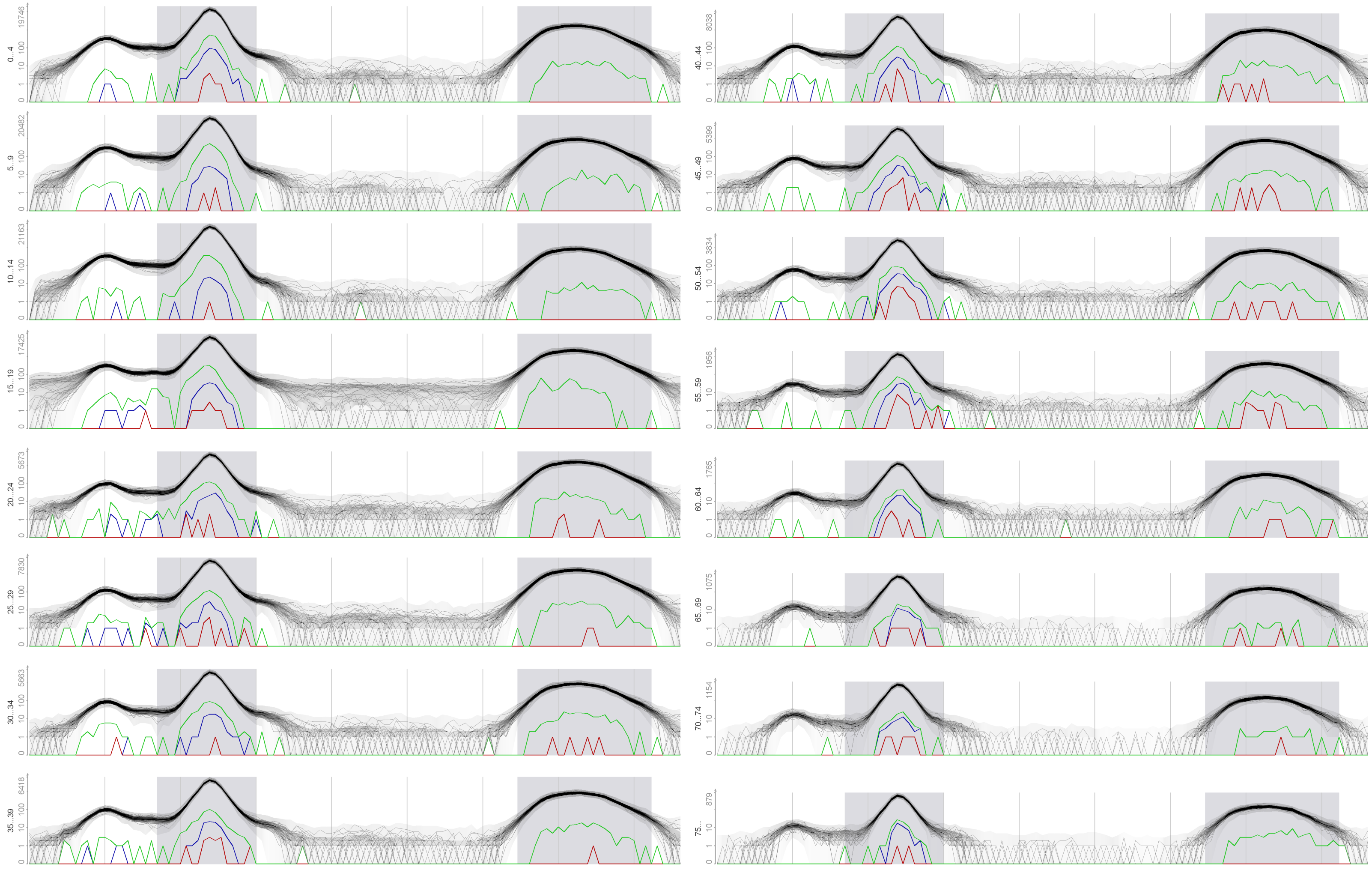

Figure 3: **Posterior distribution of the number of infections per week.** Each subplot presents a single age group. Solid lines represent the total numbers of observed cases (green), numbers of observed hospitalized cases (blue) and IC cases (red);

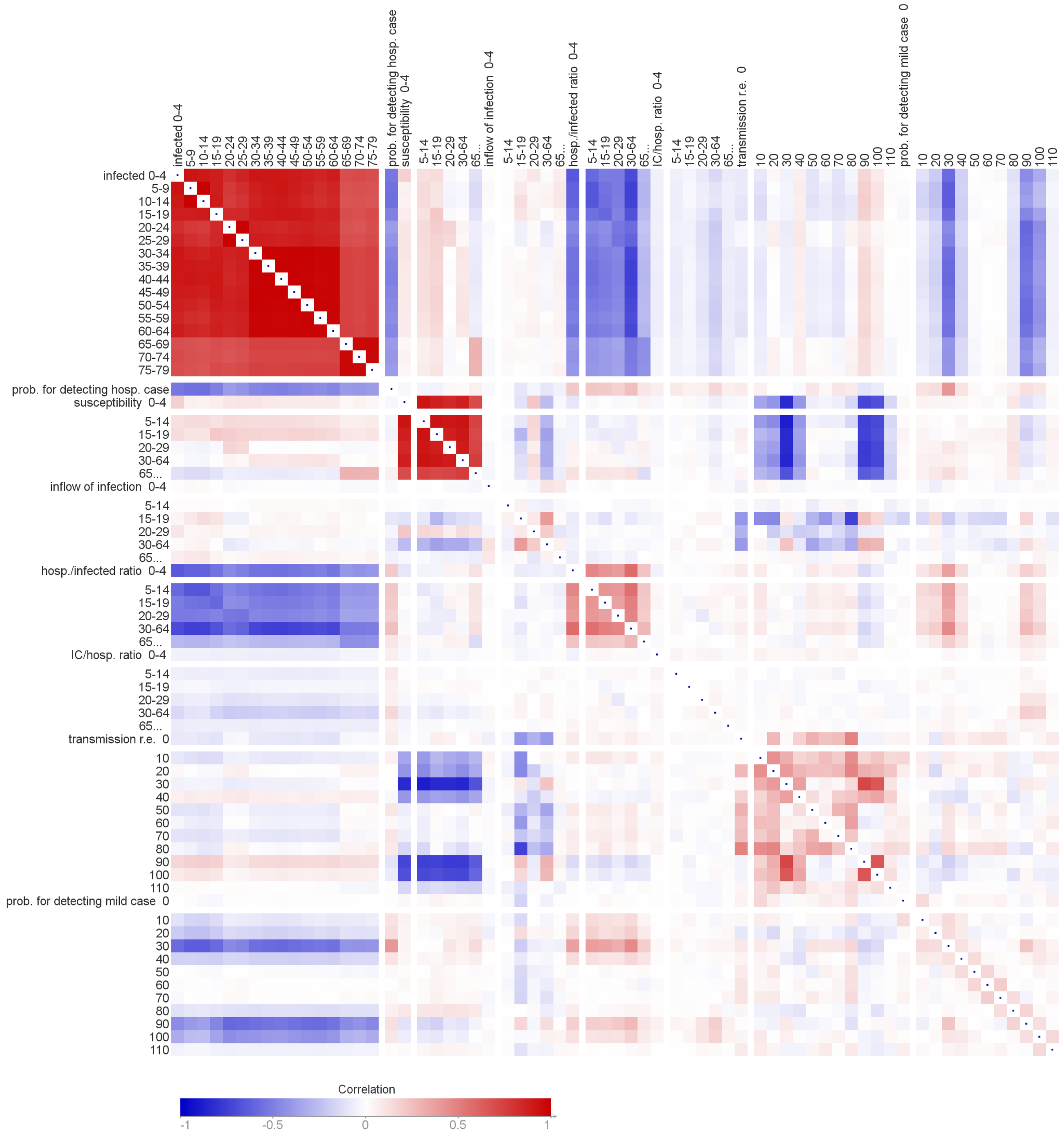

Figure 4: **Pearson Correlation matrix for some of the models unknowns.** The estimated numbers of infections and the susceptibility parameters p_a in different age groups have strong posterior correlation. There is negative posterior correlation between p_a and the transmission random effect w_t (see Fig. 5 for details). There is no strong correlation between the random effects w_t and $d_t^{(\mathrm{mild})}$.

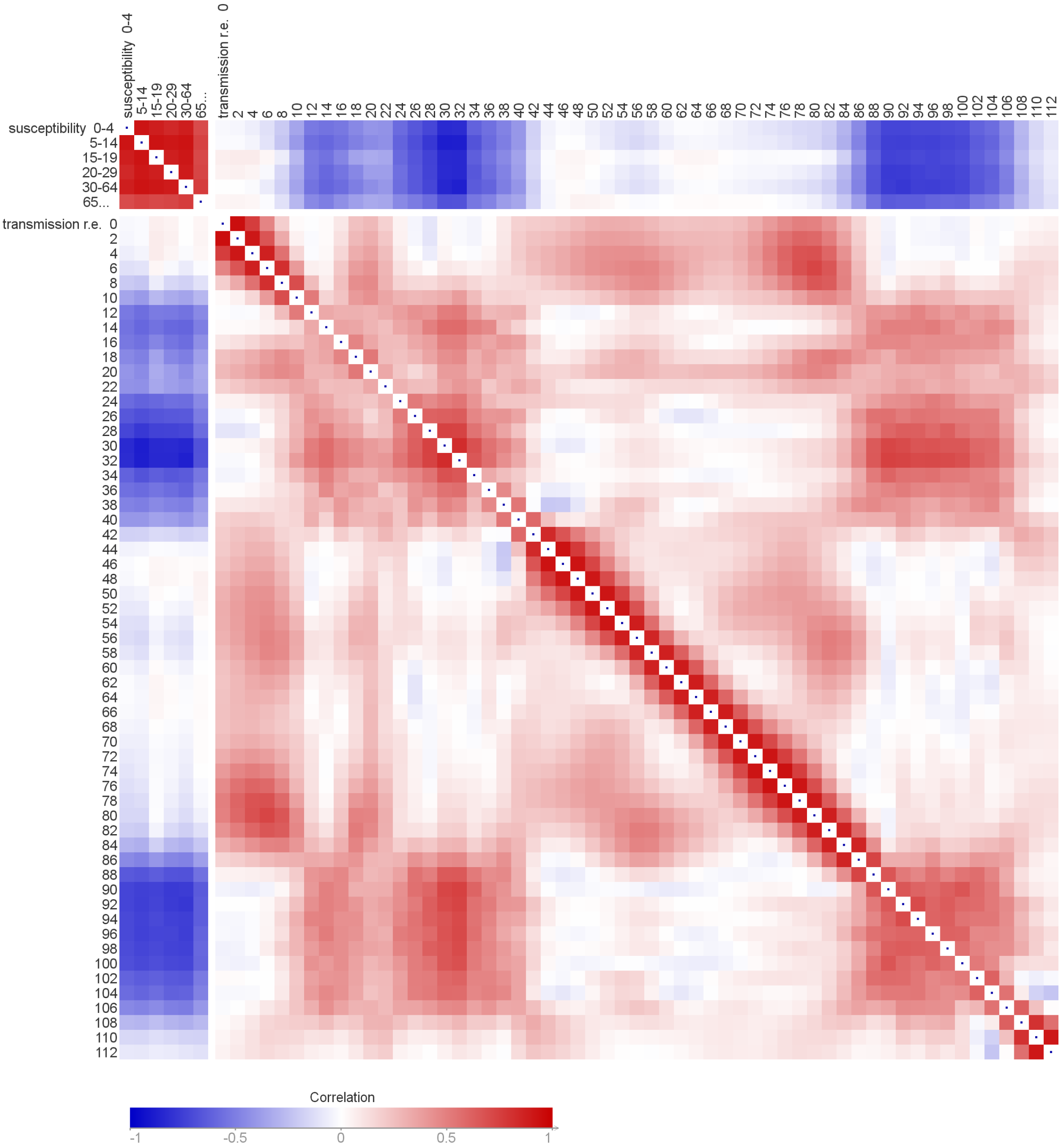

Figure 5: **Pearson Correlation matrix for the susceptibility parameters p_a and the transmission random effect w_t.** Parameters p_a show strong negative correlation with w_t around the peak of the first season ($t \simeq$ week 30) and the second season ($t \simeq$ week 90). The values of w_t are positively correlated.

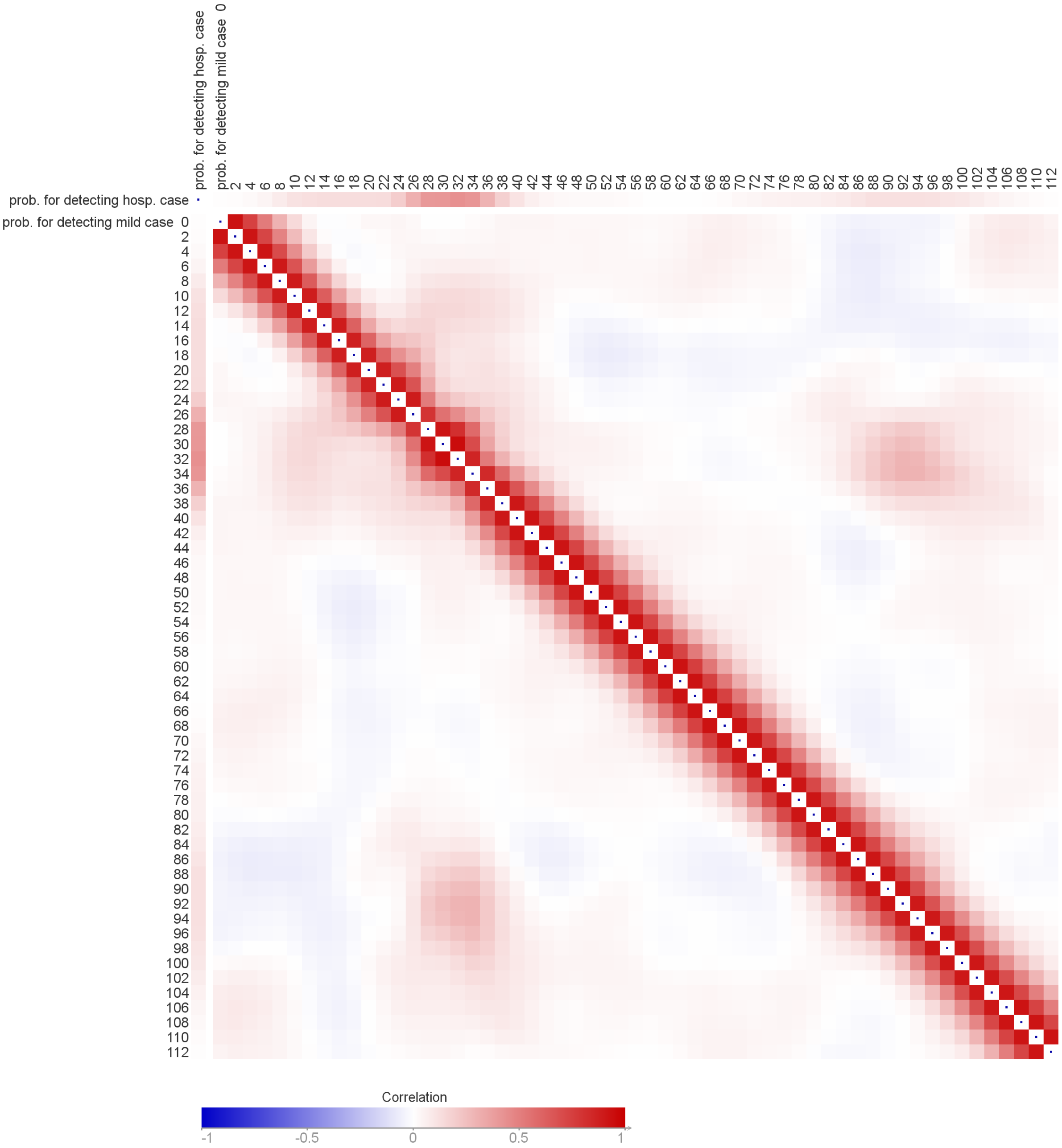

Figure 6: **Pearson Correlation matrix for the detection probabilities** $d_t^{(\mathbf{mild})}$ **and** $d^{(\mathbf{hosp})}$**.** The values of $d_t^{(\mathrm{mild})}$ for the adjacent weeks are strongly correlated, reflecting the smoothness of the process.
