# Supplementary material for: Revealing the True Incidence of Pandemic A(H1N1)pdm09 Influenza in Finland during the First Two Seasons — An Analysis Based on a Dynamic Transmission Model
Source: PLoS Comput Biol. 2016 Mar 24;12(3):e1004803. doi: 10.1371/journal.pcbi.1004803 (PMC4807082; doi:10.1371/journal.pcbi.1004803)
Supplement: S5 Appendix — (PDF) [file pcbi.1004803.s005.pdf]

Revealing the true incidence of pandemic A(H1N1)pdm09 influenza in Finland during the first two seasons

Mikhail Shubin, Artem Lebedev, Outi Lyytikäinen, Kari Auranen

Supplement 5: Additional analyses

The pictures in this Supplement illustrate the sensitivity analysis and posterior predictive checks. All distributions are visualized with more probable values represented by more concentrated color. In addition, a few samples from the distributions are shown.

1 Posterior predictive check

To validate the model, we conducted a posterior predictive check. We simulated a set of 3000 pseudo-observations using parameter values sampled from the posterior. The simulated data were then visually compared with the actual observations. The agreement was good both at the population level (Fig. 1) and at the level of single age groups (Fig. 2–4). There are no detected hospitalized non-IC cases simulated after week 60 because we assume that only mild and IC cases are recorded during this period.

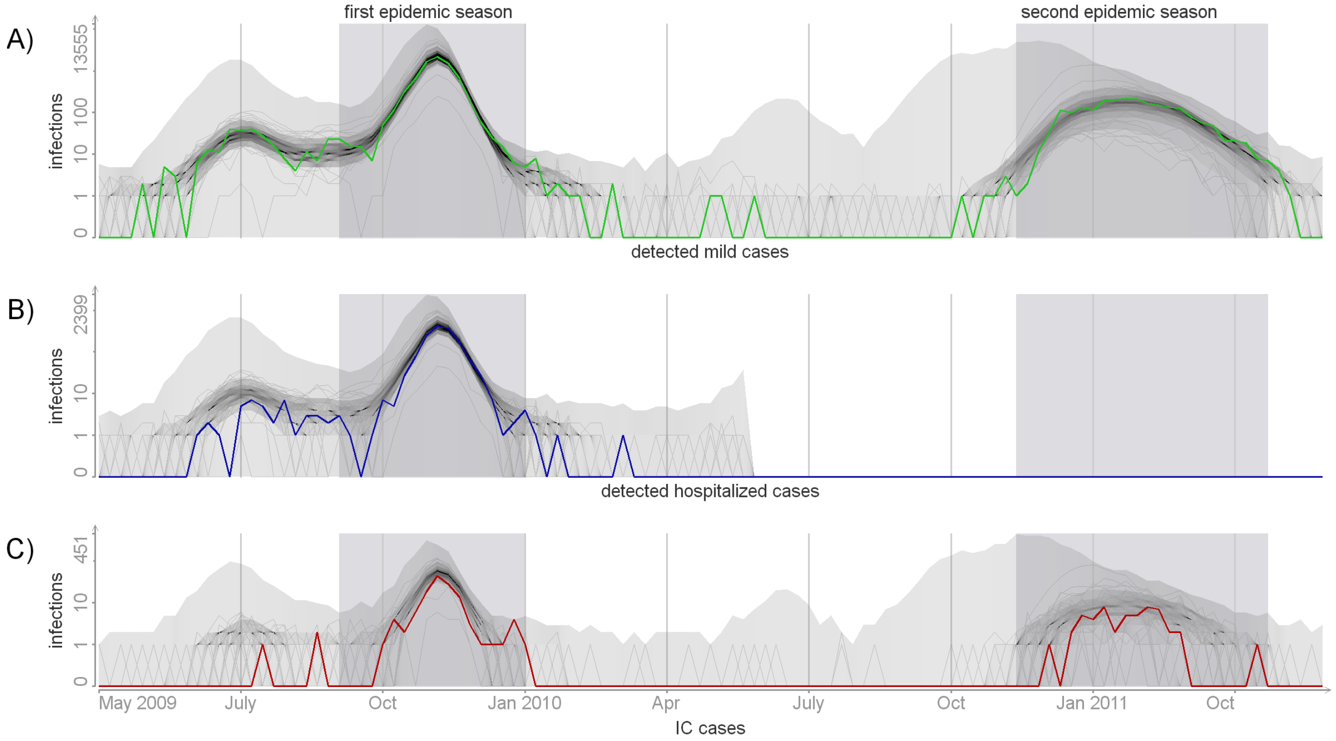

Figure 1: **Posterior predictive check.** The distributions of pseudo-observations are visualized. The solid lines indicate the observed numbers of detected cases. Panel A: mild cases; Panel B: hospitalized non-IC cases; Panel C: IC cases.

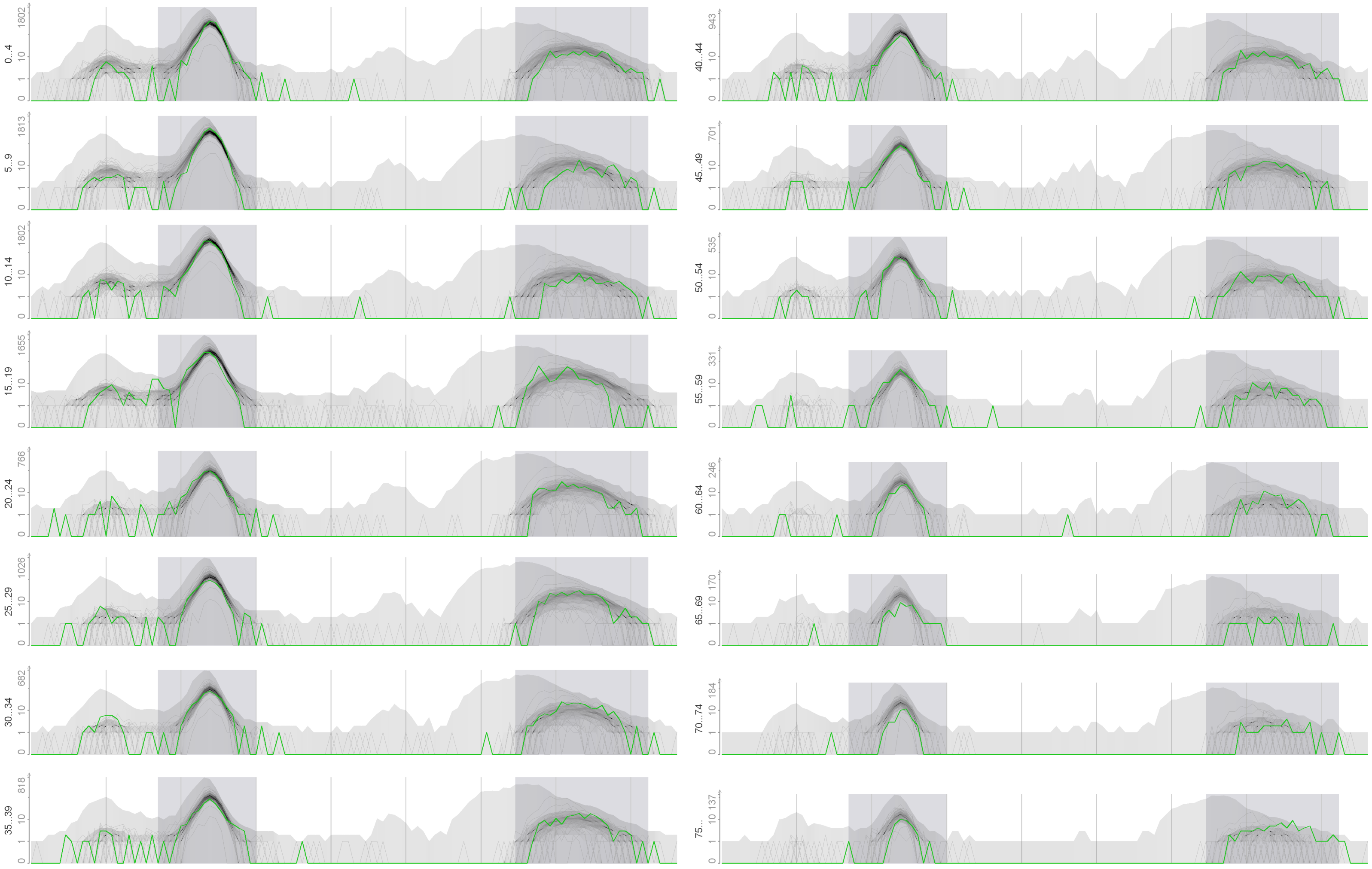

Figure 2: **Posterior predictive check by age groups; mild cases.** Each subplot presents a single age group. Coloured areas represent the distributions of pseudo-observations: Solid lines - actually observed numbers of cases.

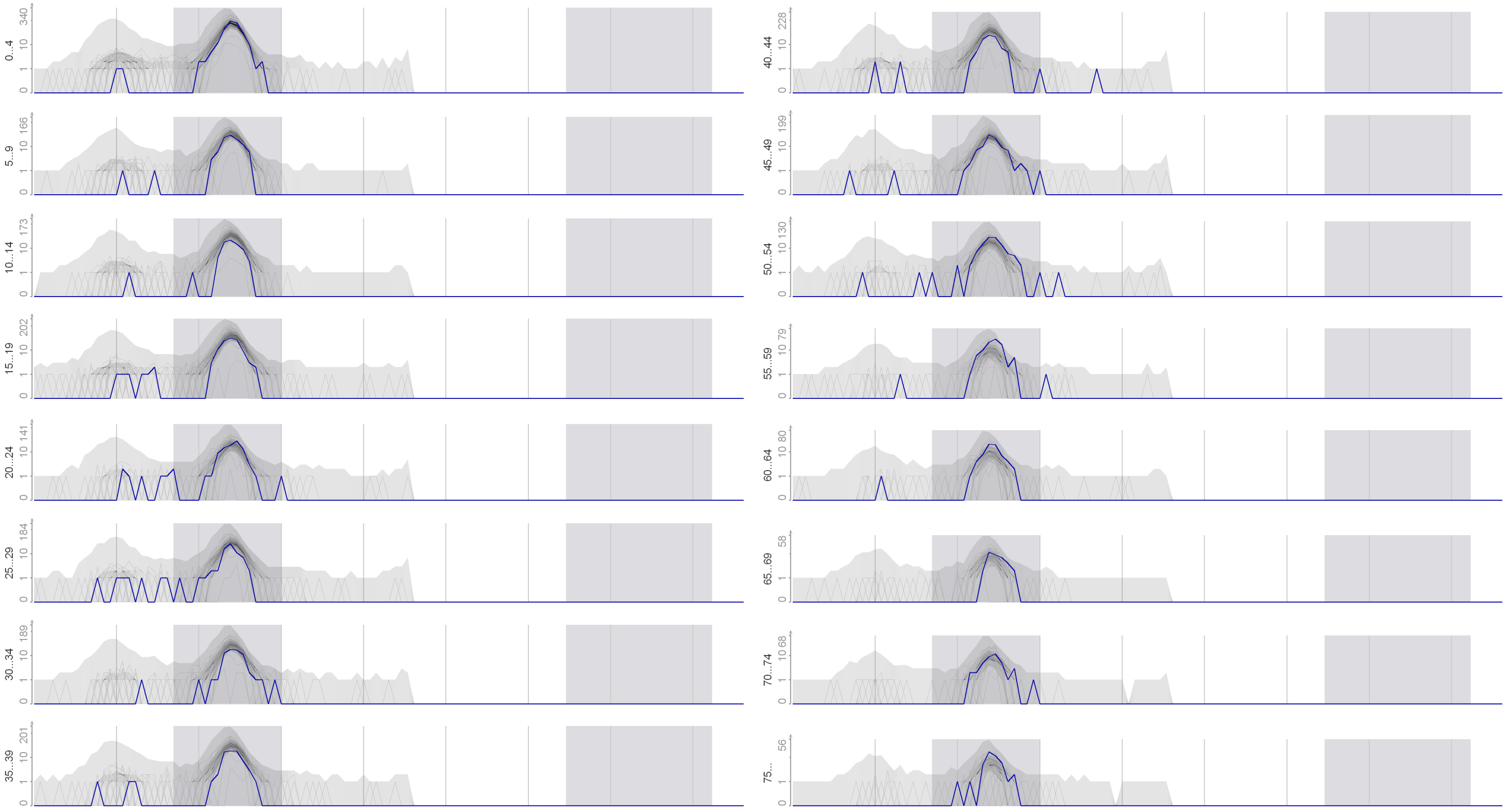

Figure 3: **Posterior predictive check by age groups; hospitalized non-IC cases.** Each subplot presents a single age group. Coloured areas represent the distributions of pseudo-observations: Solid lines - actually observed numbers of cases.

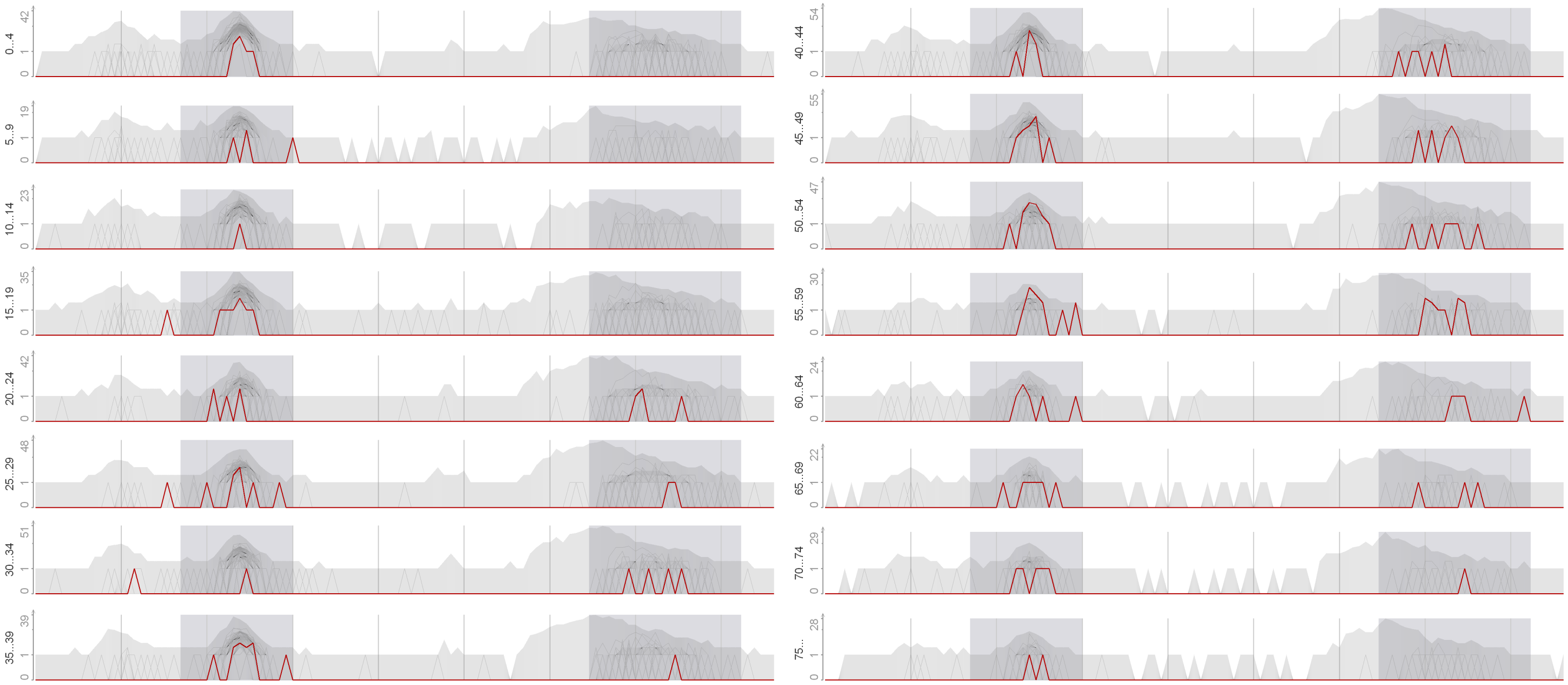

Figure 4: **Posterior predictive check by age groups; IC cases.** Each subplot presents a single age group. Coloured areas represent the distributions of pseudo-observations: Solid lines - actually observed numbers of cases.

2 Sensitivity analysis

Methods We were unable to estimate the posterior distribution for many alternative prior choices due to the computational burden of the inference method. To assess the model's robustness we compared the modes of the posteriors (i.e. maxunum *a posteriori* estimates) under different prior choices. The optimization routine (see Supplement 3) used to search for the mode was computationally much faster.

The mode of the posterior distribution differs significantly from the mean and suggests about 30% less infections. This is likely to follow from the skewness of the discrete distribution of the number of infected individuals, with the mode smaller than the mean. Nevertheless, the comparisons based on modes should be informative about how the means behave under the different prior settings.

Figures 5-9 compare the posterior distribution with the posterior mode and present the results of the sensitivity analysis.

Detection probability $d_t^{(\mathrm{mild})}$. In the base-case model (as presented in the main text), the marginal detection probability of the mild cases $d_t^{(\mathrm{mild})}$ at time point t was defined by the following prior:

$$d_t^{(\mathrm{mild})} \sim \mathrm{logitNormal}(0.01, 0.01).$$

We tried different values for the variance of the prior: 1, 0.1, 0.001 and 0.0001. We observe that $d_t^{(\mathrm{mild})}$ always stayed around the prior mean between the epidemic seasons. However, smaller prior variances led to larger detection probabilities before the outbreak of the first season and during the second season (Figure 9). This in turn led to significantly lower estimates of the incidence. the prior choice for $d_t^{(\mathrm{mild})}$ had the largest impact on the variation across the posterior estimates of the true incidence.

Severities $s^{(\mathrm{sev/inf})}$ and $s^{(\mathrm{IC/sev})}$. In the base-case model, the severities: the age specific hospitalization/infection ratio and IC/hospitalization ratio were defined by priors

$$s^{(\mathrm{sev/inf})} \sim \mathrm{logitNormal}(0.01, 0.1)$$
$$s^{(\mathrm{IC/sev})} \sim \mathrm{logitNormal}(0.1, 0.1)$$

We tried different values for the variance of both prior distributions: 1, and 0.01. We observed that smaller variances lead to lower estimates of the incidence and vice versa. The impact of the prior for $s^{(\mathrm{sev/inf})}$ was more significant.

Smoothness of the time-dependent random effects. *A priori* we assumed that time-dependent transmission random effects w_t (determining the variation in the basic reproduction number $R_{0,t}$) and detection probability for mild cases $d_t^{(\mathrm{mild})}$ are autocorrelated (but independent of each other). We implemented these priors by constructing them as samples from logistic-transformed multivariate T-dimensional normal distributions (Here $T = 113$ is number of weeks in the modelled period), with the covariance defined by kernel K:

$$K_{i,j} = \exp(-(i-j)^2/52) + 0.01 \times \mathbb{1}(i = j) \text{ for } i, j \in 0 \dots T-1.$$

The coefficient 1/52, tentatively speaking, means that w_t and $d_t^{(\mathrm{mild})}$ require a month to change. We studied the posterior modes with the coefficient substituted to 1/11 (more smooth, about 4 months to change), 1/26 (2 months), 1/104 (less smoothness, 2 weeks to change) and 1/208 (1 week). We also considered the kernel $K_{i,j} = 1.01 \times \mathbb{1}(i = j)$, i.e. no autocorrelation.

We concluded that increasing the smoothness of the prior process decreases the estimated number of infected cases. Some features of the results are robust to prior choice. The posterior values of w_t during the November 2009 are always the same (Figure 8). The posterior process w_t peaks during the initial part of both epidemic seasons. $d_t^{(\mathrm{mild})}$ always growth before the peak of the first season and during the second epidemic seasons.

Vaccine efficacy. In our study we modelled the vaccine as having a 80% chance to induce complete immunity against the infection. We assumed it took two weeks for a vaccine to take an effect. In the sensitivity analysis, we tried different values for the vaccine efficacy: 60%, 70%, 90%, 100% and different values for the vaccine delay: 0, 1, 3, 4 weeks. We found that the vaccine efficacy and delay does not influence the results of the inference a lot.

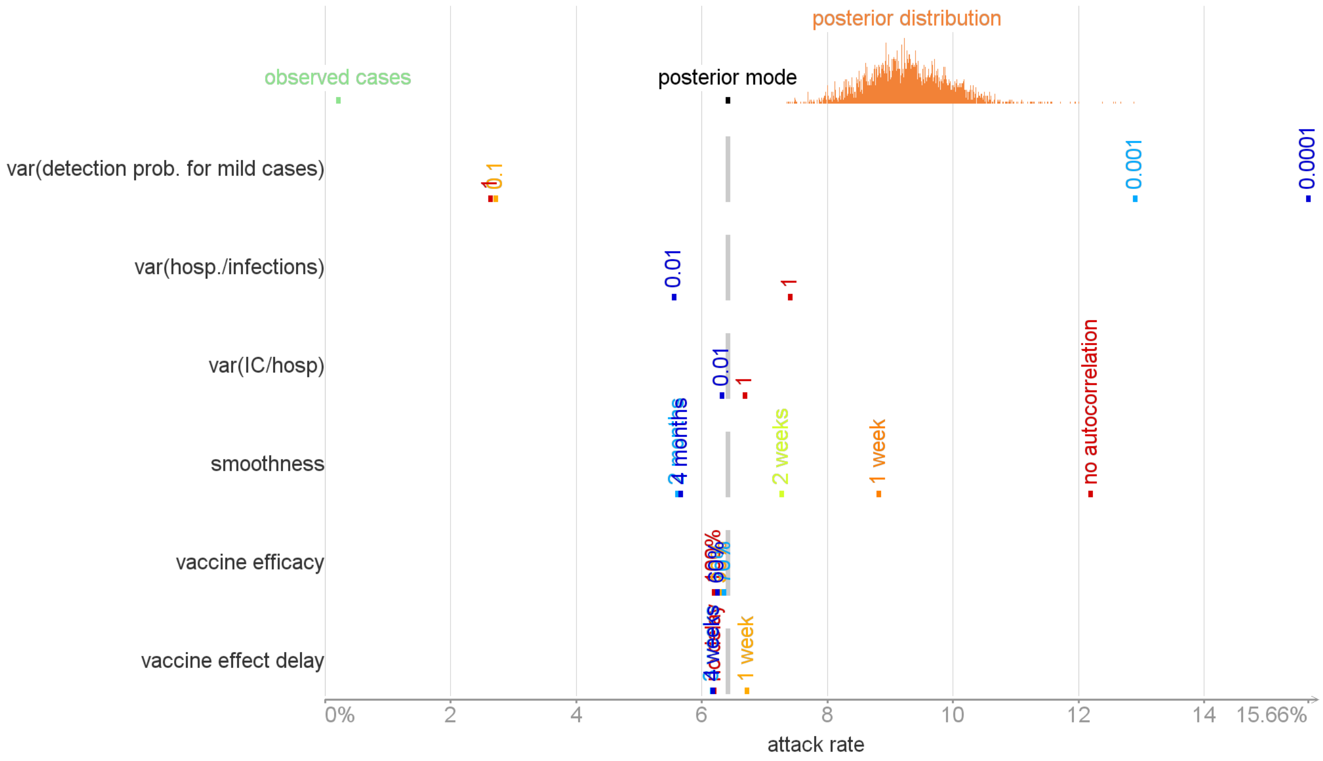

Figure 5: **Sensitivity analysis: Attack rates.** The top panel compares the posterior distribution and the mode. The rest of the panels present the modes for the different prior setting.

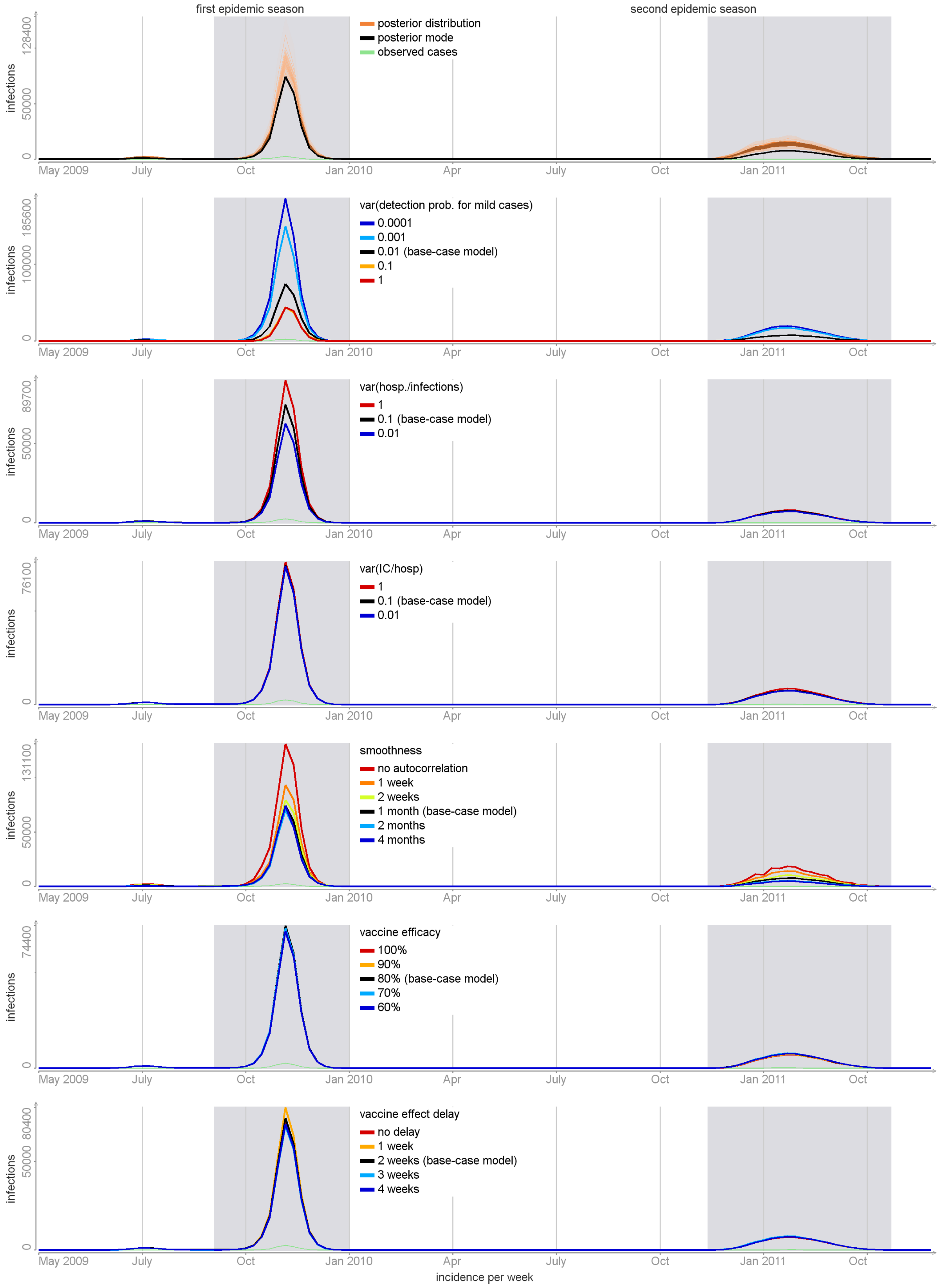

Figure 6: **Sensitivity analysis: Incidence.** The top panel compares the posterior distribution and the mode. The rest of the panels present the modes for the different prior settings.

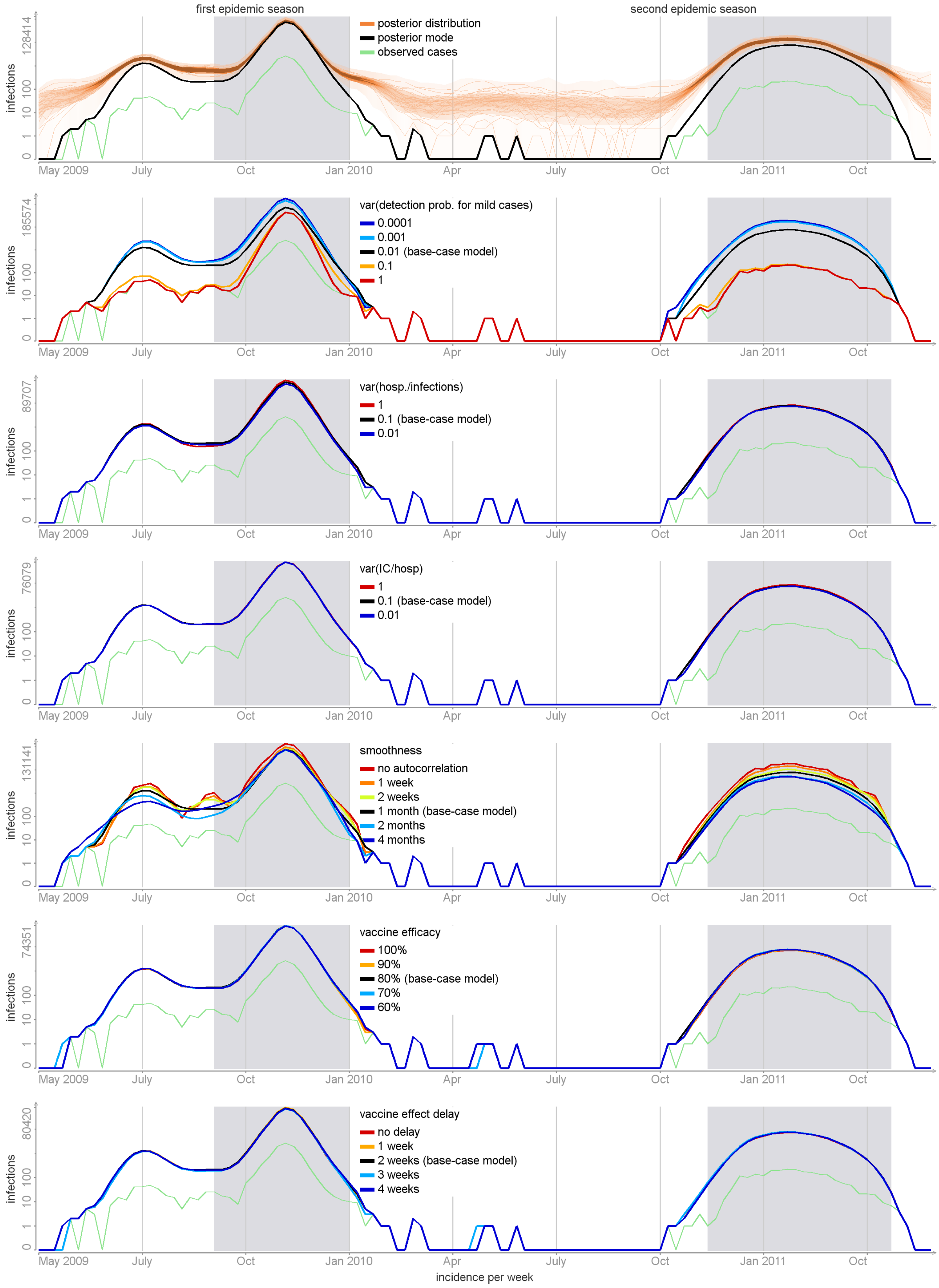

Figure 7: **Sensitivity analysis: Incidence, log scale.** The top panel compares the posterior distribution and the mode. The rest of the panels present the modes for the different prior settings.

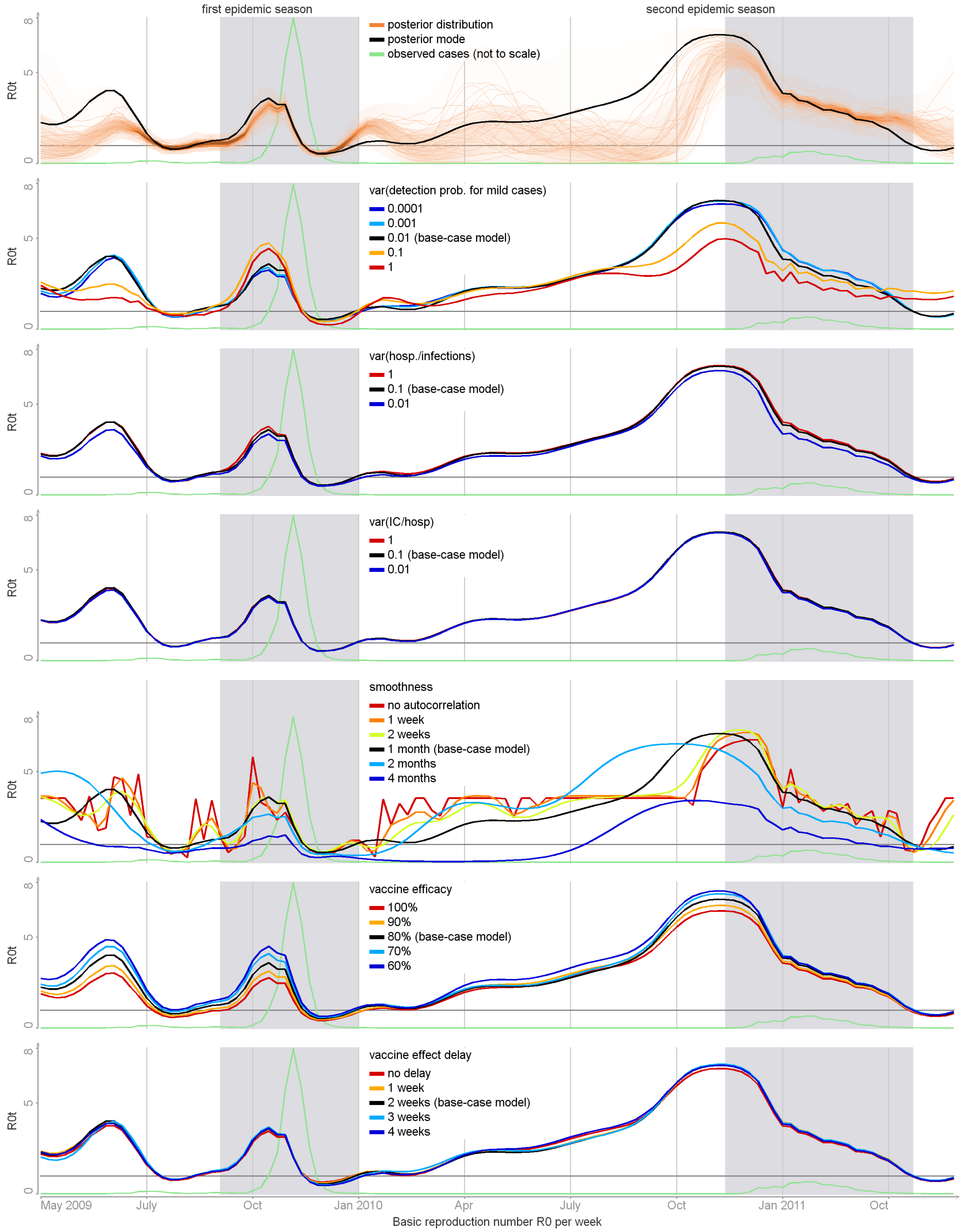

Figure 8: **Sensitivity analysis: Time-dependent basic reproduction number $R_{0,t}$.** The top panel compares the posterior distribution and the mode. The rest of the panels present the modes for the different prior settings.

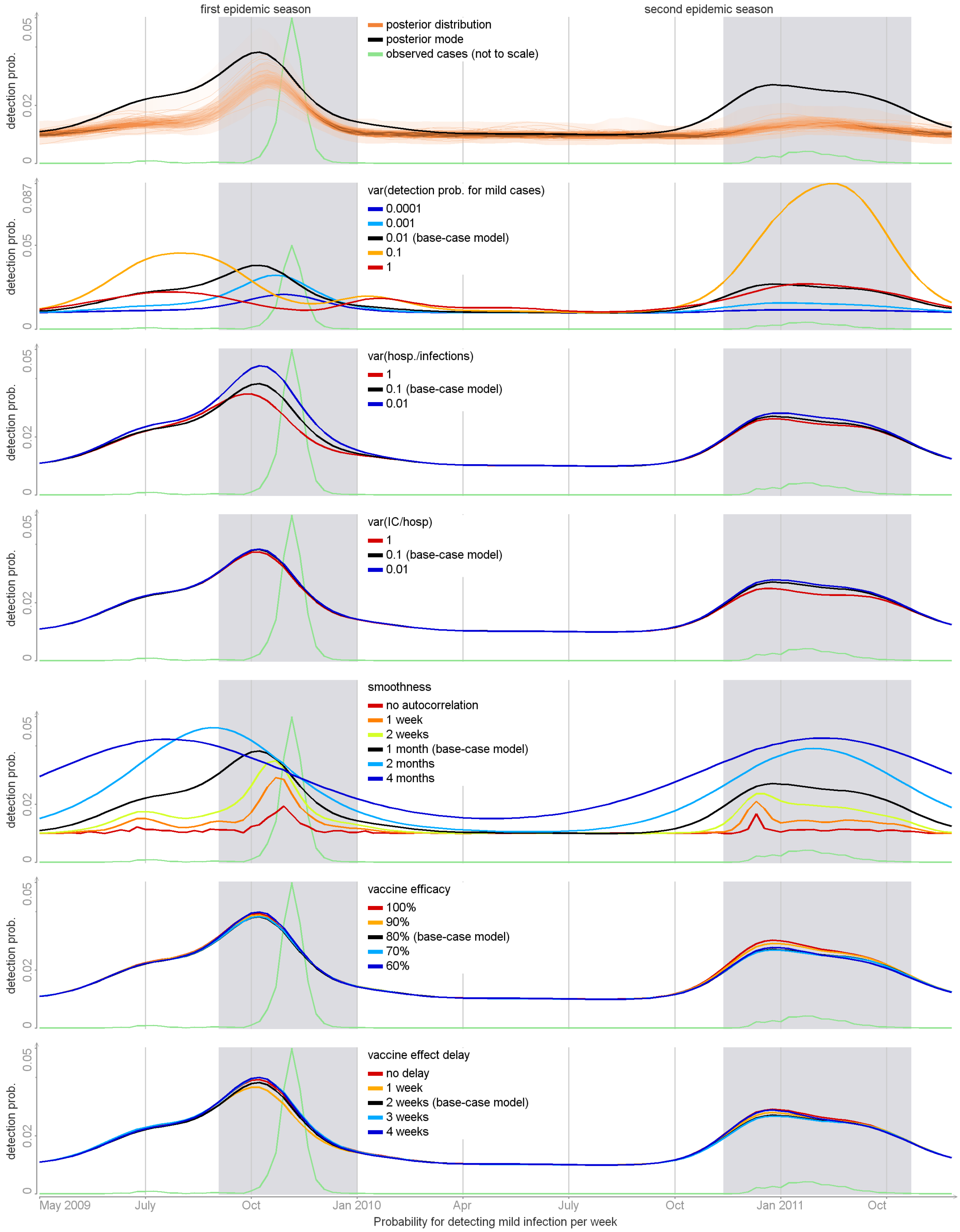

Figure 9: **Sensitivity analysis: Probability for detecting mild cases $d_t^{(\mathbf{mild})}$.** The top panel compares the posterior distribution and the mode. The rest of the panels present the modes for the different prior settings.
