# Supplementary material for: Revealing the True Incidence of Pandemic A(H1N1)pdm09 Influenza in Finland during the First Two Seasons — An Analysis Based on a Dynamic Transmission Model
Source: PLoS Comput Biol. 2016 Mar 24;12(3):e1004803. doi: 10.1371/journal.pcbi.1004803 (PMC4807082; doi:10.1371/journal.pcbi.1004803)
Supplement: S6 Appendix — (PDF) [file pcbi.1004803.s006.pdf]

Revealing the true incidence of pandemic A(H1N1)pdm09 influenza in Finland during the first two seasons

Mikhail Shubin, Artem Lebedev, Outi Lyytikäinen, Kari Auranen

Supplement 6: Continuous and discrete-time SIR models

In this supplement, we show that the discrete-time model used in this study behaves similarly to the standard continuous-time SIR model with the same R_0. In particular, both models lead to the same final size (the total number of individuals infected during the epidemic) (Diekmann and Heesterbeek, 2000) and similar distributions of infection over time.

The SIR model in a homogeneously mixing population (Diekmann and Heesterbeek, 2000) is defined by the system of differential equations:

$$
\begin{aligned}
S' &= -SI\alpha, \\
I' &= \quad SI\alpha - I\beta, \\
R' &= \quad I\beta.
\end{aligned}
$$

Here S, I and R are the numbers of susceptible, infectious and removed individuals, α is the infection rate (the rate of infectious contacts one infected individual exerts per individual in the population) and β is the recovery rate. The total population size $N(t) = S(t) + I(t) + R(t)$ remains constant over time. The basic reproduction number R_0 is defined as the number of secondary infections caused by one infective individual in a completely susceptible population. For the SIR model, $R_0 = N\alpha/\beta$.

As the SIR system does not have an analytical solution, we approximate it numerically as follows:

$$
\begin{aligned}
S(t + \Delta t) &= S(t) - S(t)I(t)\alpha\Delta t, \\
I(t + \Delta t) &= I(t) + (S(t)\alpha - \beta)I(t)\Delta t, \\
R(t + \Delta t) &= R(t) + I(t)\beta\Delta t.
\end{aligned}
$$

Here Δt is the time step of the approximation. The approximation is accurate when $\Delta t \to 0$.

We define the corresponding deterministic discrete-time SIR model as following:

$$\begin{aligned} I(t+1) &= S(t) \times \left(1 - \left(1 - \frac{p}{N}\right)^{CI(t)}\right); \\ S(t+1) &= S(t) - I(t+1); \\ R(t+1) &= R(t) + I(t). \end{aligned}$$

Here p is the probability for an individual to become infected per contact and C is the number of contacts per time step. The basic reproduction number of this model can be approximated with $R_0 = pC$.

The stochastic version of discrete-time SIR model samples the number of infections using the binomial distribution:

$$\begin{aligned} I(t+1) &\sim \text{Binom}\left(S(t); 1 - \left(1 - \frac{p}{N}\right)^{CI(t)}\right), \\ S(t+1) &= S(t) - I(t+1), \\ R(t+1) &= R(t) + I(t). \end{aligned}$$

Simulations were repeated for several values of N, R_0, α, β, p and C such that $R_0 = N\alpha/\beta = pC$. Figure 1 shows the results for N =100 000, $R_0 = 2$, $\alpha = 1.5/N$, $\beta = 0.75$, $p = 1$, $C = 2$. We observe that the results of continuous- and deterministic discrete-time models are similar in the time dimension and almost identical in the (S, I) space. This means that both models lead to the same final size of the epidemic (the number of removed individuals when $t \to \infty$). Stochasticity adds additional noise to the time dimension and the probability that the epidemic would die out before starting the outbreak. However, if the number of infections reaches some threshold, the model follows closely its deterministic version.

Changing α and β while keeping R_0 intact scales the trajectory of the SIR model on the time axis, but does not change its behaviour in the (S, I) space. Changing p and C while keeping R_0 intact does not change the behaviour or the discrete-time SIR.

Similar tests with similar results were performed for the SIR model with two subpopulations. In the research we applied the same model with 16 sub-populations (age groups). In addition, the actual model involved time-dependent modulation of the reproduction number by random effect w_t, the inflow of infections q and the vaccination.

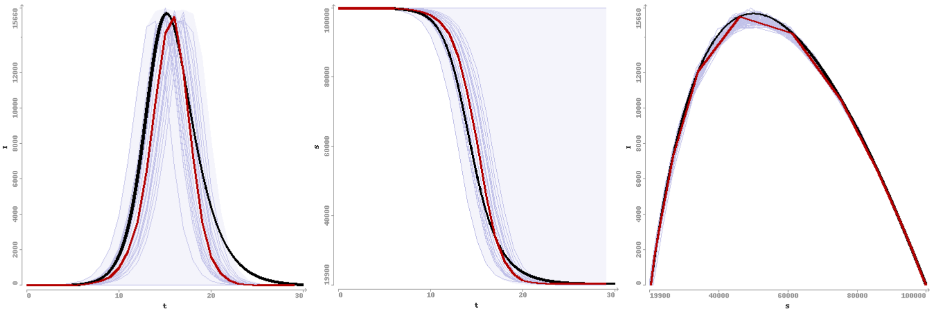

Figure 1: Trajectories of the continuous-time SIR model (black line), deterministic discrete-time SIR (red line) and the stochastic discrete-time SIR (samples are shown with the blue lines).

References

Diekmann, O. and Heesterbeek, J. A. P. (2000). *Mathematical epidemiology of infectious diseases : model building, analysis, and interpretation*. Wiley series in mathematical and computational biology. John Wiley, Chichester, New York.
